# Reject common measures like *d*′ and *Corrected Recognition*, embrace *d*_a_: Simulation explorations of single- and multi-point recognition measures of sensitivity

**DOI:** 10.3758/s13428-026-03130-w

**Published:** 2026-09-03

**Authors:** Adva Levi, Raz Danino, Caren M. Rotello, Yonatan Goshen-Gottstein

**Affiliations:** 1 School of Psychological Sciences, Tel Aviv University, Tel Aviv-Yafo, Israel; 2 Department of Psychological and Brain Sciences, University of Massachusetts Amherst, Amherst, MA USA

**Keywords:** Recognition memory, Sensitivity, Bias, Signal-detection theory, Measurement crisis, Monte-Carlo simulations

## Abstract

In this article, we address the high prevalence of false discoveries in recognition memory research. Using Monte Carlo simulations, our goal was to find a valid measure of performance that reliably separates the contribution of *sensitivity* (accuracy) from that of *bias*. Relevant to myriad tasks, most notably old–new recognition memory, the simulations revealed that common measures confound sensitivity with bias, a finding termed the “measurement crisis.” As a solution, we propose a version of d-sub-a (*d*_a_). We ran comprehensive simulations to evaluate the validity of sensitivity measures, including *P*_r_ = HR − FAR, *A*′, *d*′, and *AUC*_g_, in addition to *d*_a_. Memory “signals” were randomly sampled from lure and target distributions. Sensitivity measures generated from iso-sensitive conditions that differed in bias were compared using *t*-tests, across thousands of simulations. For bias-independent measures, the rate of significant results should be 5%. We manipulated several parameters, including the form of the distributions (i.e., from three prominent models of recognition–memory: unequal variance signal detection [UVSD], double-high threshold [2HT], dual-process signal detection [DPSD]), the distance between their means, their relative variance, the placement of response criteria, the sample size, and the number of simulated trials. Results demonstrated that under most experimental scenarios, only *d*_a_ was unaffected by changes in bias. In contrast, all common measures typically exhibited alarmingly high false discovery rates, exceeding 5%. The rates rose to 100% with larger sample sizes and a large number of trials. These findings indicate that *d*_a_ warrants serious consideration as the default measure of sensitivity.

**Supplementary Information:**

The online version contains supplementary material available at https://doi.org/10.3758/s13428-026-03130-w.

## Introduction

Consider the following scenario: Researchers test patients on their memory performance compared to aged-matched controls, using a recognition memory test. They conclude that patients’ memory is significantly impaired. Treatment plans ensue, emphasizing memory improvement. But what if patients’ ability to encode and retrieve items from memory is like that of the control group, and they only differ in bias, a higher (or lower) tendency to classify items as “old”?

This scenario demonstrates the concerns that underlie this article—the high prevalence of false discoveries in recognition memory (cf., Open Science Collaboration, 2015)—specifically, due to invalid measurement of participants’ performance. The crux of the matter is that commonly used accuracy (more formally, *sensitivity*) measures in recognition memory are confounded with bias. Therefore, even if experimental conditions are equal in sensitivity, standard sensitivity measures may nevertheless erroneously show differences across the conditions if the conditions differ in bias.

To bring home the point, a recent article described a “crisis of measurement” facing the field of recognition memory (Brady et al., 2022). This crisis entails the possibility that many of the results reported in the memory literature represent false discoveries, creating the need for a truly bias-independent measure for recognition research. In this article, we seek to promote a solution to the measurement crisis that will equip recognition memory researchers with a valid measure of performance in the form of a *scalar value of sensitivity*. To this end, we will demonstrate the properties of a little-known sensitivity measure and show it to be free of the bias confound under almost all circumstances.

The idea that problematic measures mediate discoveries that may be false has been documented in many research domains (for a detailed description, see Rotello et al., 2015). For example, in the legal domain, research first demonstrated an enhanced ability to distinguish guilty from innocent suspects in eyewitness lineups when the suspects were presented for inspection sequentially, one at a time, as compared to when suspects appeared simultaneously. This sequential superiority effect was discovered when lineup accuracy was indexed with the *diagnosticity ratio*^1^. It turned out, however, that the diagnosticity ratio cannot be used as an index of sensitivity, because it confounds sensitivity with bias (Mickes et al., 2012). Other effects have also been found to infer a sensitivity change across conditions that only differ in bias, including the “revelation effect” (Verde & Rotello, 2003, 2004a), the effect of negative emotions in memory (Dougal & Rotello, 2007; Kapucu et al., 2008), the “belief bias effect” in the domain of reasoning, the “shooter bias” in the domain of social psychology, and maltreatment referrals in the domain of child welfare (Rotello et al., 2015).

While the current investigation is focused on recognition memory, it is likely relevant to a family of tasks known as “single-interval tasks” (Hautus et al., 2022). Such tasks are ubiquitous across all domains of cognitive research, including perception (e.g., visual discrimination task), attention (e.g., spatial cueing paradigm), decision-making (e.g., multi-element averaging task), language (e.g., semantic relatedness or lexical decision task), and metacognition (e.g., judgment of learning task). These tasks comprise single items that are presented at test for a binary judgment (e.g., seen–unseen; left–right) for which there is an objectively correct answer. Old–new recognition falls into this category because participants are asked to judge, at test, whether each item is an “old” item that had been previously presented at study (i.e., a target) or whether it is a “new” item that had not been presented at all (i.e., lure). Conclusions drawn from this article regarding the recognition memory task are likely relevant to other single-interval tasks.

In old–new recognition, researchers aim to find a difference in performance between two (or more) test conditions (e.g., shallow as compared to deep encoding, Craik & Tulving, 1975). The dependent variable, *sensitivity,* refers to how sensitive participants are to the differences between the mnemonic signals associated with targets as compared to those of lures (Hautus et al., 2022). Participants can correctly judge an item as a target (hit: the item was indeed a target) or do so erroneously (false alarm: the item was in fact a lure).

Though the latent variable that researchers are interested in is sensitivity, *bias*— the proclivity for one choice over the other—also impacts participants’ responses (Hautus et al., 2022). Participants with a more liberal bias will have a higher rate of “old” responses, whereas participants with a more conservative bias will have a lower rate of “old” responses, irrespective of whether the item is a target or a lure.

There is no doubt that a valid measure of sensitivity must be independent of bias. At an operational level, if two conditions do not differ in sensitivity but differ in bias, a valid measure should erroneously indicate a significant sensitivity difference between the two conditions only 5% of the time due to Type I errors (here and elsewhere, we assume an *α* level of 5%). We say that the measure is “bias-independent”^2^ when the Type I error rate is approximately 5% under null hypothesis scenarios.

The idea that a sensitivity measure should be bias-independent is part of many researchers’ tacit (and for some, explicit) knowledge. To illustrate, most researchers^3^ would shy away from inferring changes in sensitivity between two conditions due only to differences in hit rate (HR, the proportion of old responses to targets; e.g., 90% and 60%, under deep and shallow encoding, respectively). It is rather obvious that HR is not bias-independent; differences in HR between conditions can reflect changes in sensitivity, in bias, or in both. Unintuitively, in this article we will demonstrate that even when HRs are balanced or “corrected” with false alarm rates (FARs; the proportion of old responses to lures), most resultant sensitivity measures are not bias-independent under most circumstances.

Indeed, numerous measures of sensitivity have been proposed with the goal of obtaining a measure that indexes sensitivity irrespective of bias. Different measures use the operating point—a pair of [FAR, HR] observations—and balance the magnitude of HR with that of the FAR in different ways. Because only a single operating point is used, the family of such sensitivity measures is called *single-point sensitivity measures* (SPSMs). Different SPSMs are premised on unique assumptions regarding the population from which the data are sampled (for details, see “measurement model”) 4.

The task of finding a valid measure of sensitivity for recognition memory is not trivial. A core epistemological challenge is that “sensitivity” has no objective external criterion to which the measurement can be compared. When attempting to test the validity of a given measure, we aim to demonstrate that a difference in sensitivity did or did not occur. However, we can only identify differences in sensitivity (or lack thereof) through the very sensitivity measures whose validity we are testing.

One path to avoid such circularity—the path taken in the article—is to repeatedly run *simulated* experiments in which the true sensitivity is known, and gauge Type I error rates (T1ERs) across the experiments. By simulating artificial data where the population parameters are known, a measure’s values can be calculated to evaluate the extent to which false significant differences emerged under these parameters. Critically, this process entails no circularity and is independent of any particular measure, creating an external criterion that is missing in empirical research.

Given that thousands of experiments have been run using recognition memory, can one safely assume that indices of sensitivity have been validated multiple times through such simulations? Alas, only a handful of simulations have been executed, with none uncovering a valid measure of sensitivity. Specifically, Monte Carlo simulations (Schooler & Shiffrin, 2005; Rotello et al., 2008) convincingly demonstrated a bias confound in measuring recognition memory performance when using any of the standard sensitivity measures (e.g., *Percent* *correct*, *P*_r_ = HR – FAR, *d*′, *A*′). These simulated experiments demonstrated how changes in bias across conditions often masquerade as changes in sensitivity. Here, we have extended these simulations in several ways and present a possible solution to the measurement crisis—our primary goal (see Appendix A for all of the data we collected through simulations). We argue that the little-known measure, d-sub-a (denoted *d*_a_), offers the characteristics that provide a remedy to the crisis.

The current research is modeled after the simulations of Rotello and her colleagues (2008), which included a larger number of parameters and a larger number of trials than preceding work. Rotello et al. simulated two iso-sensitive aniso-bias conditions and indexed sensitivity using different measures. They then compared the sensitivity of the two conditions with *t*-tests, to assess whether Type I errors would occur at rates higher than 5%. The simulations tested the validity of the different measures under varying parameters, including the forms of lure and target distributions, the manipulation strengths (i.e., different distances between the means of the lure and target distributions), the criterion placements, the sample sizes, and the number of trials. Across the variety of parameters and across a thousand iterations, the rate of Type I errors was tallied. The results revealed that by and large, all common measures used for demonstrating different levels of sensitivity in tasks such as recognition are *not* bias-independent^5^.

A notable shortcoming of the Rotello et al. (2008) simulations was that no alternative was advanced as a potential valid measure in recognition memory. The primary goal of this article was to rectify this very shortcoming. Alongside this main goal, we will attempt to replicate the original Rotello et al. (2008) simulations. Such a replication is important for at least two reasons. First, like the need for a replication of empirical experiments, simulations too must be independently replicated (Lohmann et al., 2022). Were there mistakes in the code? Did the simulations sample the parameter space appropriately? If we wish to consider the implications of the simulations—high rates of Type I errors—their results must first be shown to replicate.

Second, the results of the original simulations did not receive the attention they deserved from the scientific community. At the time the current article was written, the simulations had been cited only 110 times. Researchers continue to use invalid measures, even today. To gauge the frequency with which different sensitivity measures are used, we reviewed hundreds of articles published by Springer Nature in the 7 years spanning 2018–2024 that reported using old–new recognition memory tasks (including *Memory & Cognition*, *Nature Human Behaviour, Scientific Reports*, *Psychonomic Bulletin & Review*, *Cognitive Affective and Behavioral Neuroscience*, *Cognitive Research: Principles and Implications*, *Nature Communications*).

**Fig. 1:**
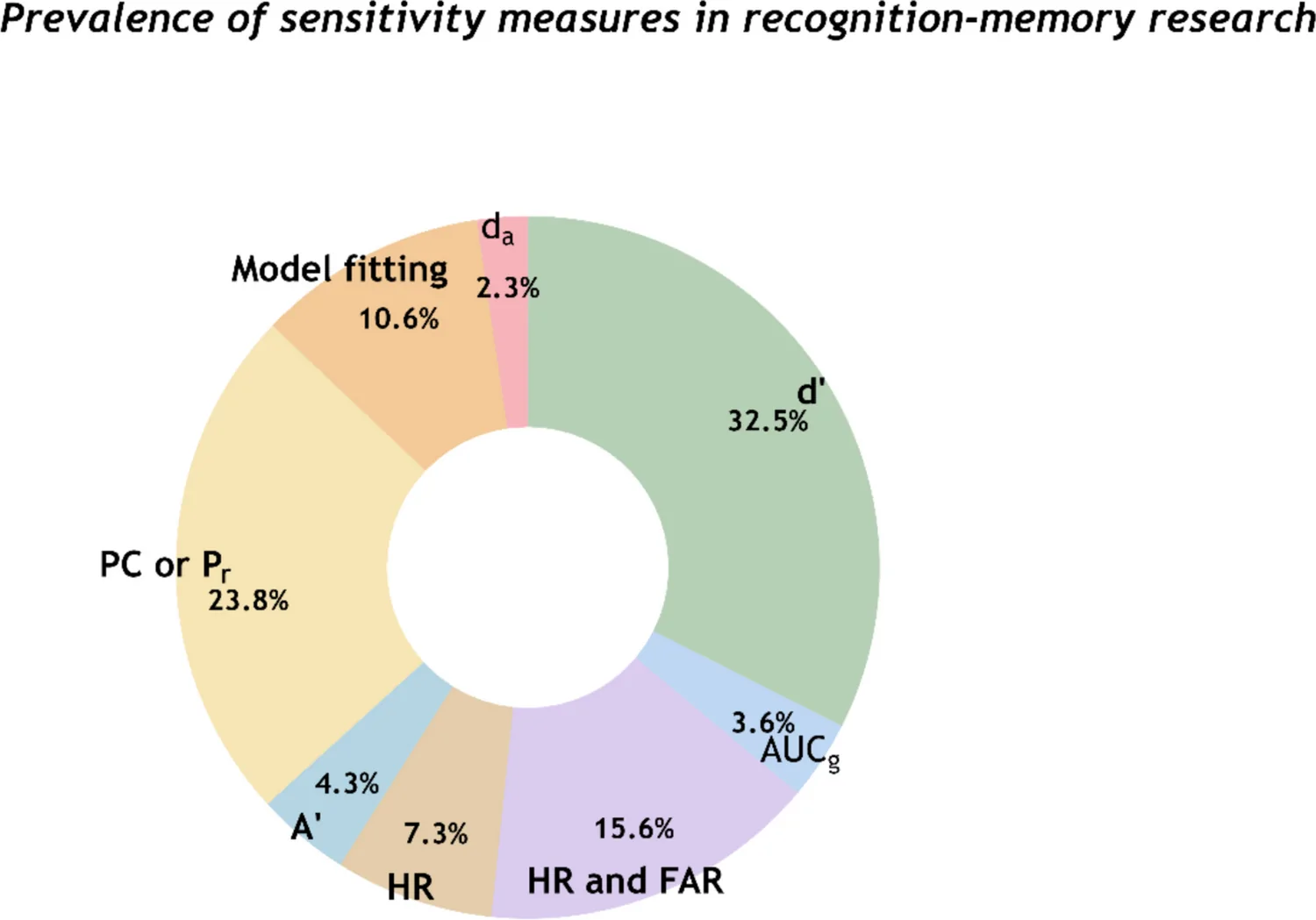
Frequency of use of the different sensitivity measures in hundreds of old–new recognition memory articles published in 40 Springer Nature journals during the 7 years spanning 2018–2024. In total, 253 articles were included in this review. Articles that analyzed their recognition results by means of more than one measure were tallied for each measure separately, for a total of 302 recognition measures. All articles reported at least one of nine different measures or methods to index sensitivity. We divided these measures into eight categories. The frequency of articles that used PC (percent correct) and *P*_r_ (= HR − FAR) was 12.9% and 10.9%, respectively. PC and *P*_r_ appear in the figure in a single category (with a combined frequency of 23.8%) because they are linear transformations of each other. Both measures assume a threshold model. Also, almost all the articles that used model fitting assumed a threshold model as their data-generating model

In total, 253 recognition memory articles were reviewed, each reporting at least one of the nine measures depicted in Fig. 1. Because several articles used more than one measure for indexing sensitivity, the total number of measurements in these articles was 302. The use of so many different measures in the literature, and the high frequency of *Percent* *correct* (PC), *P*_r_, and *d*′, is intriguing, if not troubling, given that none of the measures has been validated. Indeed, measures identified in our review, including PC, *P*_r_, and *d*′, were found to be demonstrably invalid under a large number of experimental scenarios^6^.

It is quite apparent, therefore, that the influence of the Rotello et al. simulations on the field has been negligible. This is bad news for science when considering the far-reaching implications of their results, which raised serious questions about the validity of all common measures of sensitivity. Let us be blunt: Many recognition studies that used standard measures and reported differences in sensitivity may have reached conclusions that are not supported by their data (see “Guidelines for evaluating previous recognition research” in the General discussion for a few limited exceptions).

It seems that the research community has only been stirred to reflect on research practices following the “replication crisis” (Open Science Collaboration, 2015). The alarmingly low rates of replicable results caused an influx of articles describing the ailments of current procedures and possible cures (e.g., Smith & Little, 2018). New journals were created (e.g., *Advances in Methods and Practices in Psychological Science*; existing journals, including *Psychological Science* and *Nature Human Behaviour*, also made adjustments and introduced “Registered Reports”), and scientific conferences devoted entire sections to addressing issues of methodology (e.g., methodology sections in the Annual Meeting of the Psychonomic Society; the founding of the Society for the Improvement of Psychological Science, which holds annual conferences to improve methods and practices in psychological science). Today’s researchers are probably better prepared to process the alarming news that the original simulations bear. It is time, therefore, that they be replicated. To reiterate, in addition to our primary goal of promoting *d*_a_, we re-examined whether standard sensitivity measures are indeed confounded with bias. In addition, we added to our test bed of indices a sensitivity measure that is often used in other disciplines (e.g., machine learning)—the area under the ROC curve (*AUC*_g_; see below for a detailed description of this non-parametric measure).

In our current investigations, we ran Monte Carlo simulations generating thousands of experiments that compared iso-sensitive conditions differing in bias. We explored which of the different measures of sensitivity (*P*_r_, *A*′, *d*′, tested in the original simulations; *AUC*_g_ and, of course, *d*_a_, both tested here for the first time) would show erroneous significant differences only at a rate of 5%. To anticipate the results, we replicated the previous findings that all the previously reported measures, as well as *AUC*_g_, confound sensitivity and bias. In contrast, one sensitivity measure fared quite well—*d*_a_. We thus offer *d*_a_ as a possible solution to the measurement crisis.

### The roots of the measurement crisis

To understand the roots of the measurement crisis, we remind readers of the psychometric concepts of reliability and validity. *Reliability* relates to the ability to repeat experiments and obtain similar results. The notion of replicability entails the idea that results must be highly reliable. A measuring tape is reliable for measuring the lengths of different tables but would be less reliable (or not reliable at all) if measuring the widths of different strands of hair. *Validity* describes the extent to which changes in the values of the dependent variable are related to the (typically latent) construct it is supposed to measure. Thus, if a measuring tape were to be used to measure the color of a table, the values read from the measuring tape would not capture the hue of the table. This is even though these values might be highly reliable. A measure or test may have a high level of reliability yet not be valid.

As a generality, the replicability crisis has focused on enhancing reliability (e.g., on running high-powered experiments and using large sample sizes; Open Science Collaboration, 2015). Importantly, highly replicable results do not necessarily represent true discoveries. *If a study is reliable—easily replicated—but its conclusions have low validity, it may likely constitute a false discovery and would thus steer the research community away from discovering true scientific effects*, rather than advancing our knowledge. This is not merely a dramatic hypothetical: Rotello et al. (2015) provided two examples of research domains in which multiple decades of effort chased false discoveries that stemmed from invalid measures.

Recognition memory research seems to not be valid, as demonstrated by the high rate of false discoveries in simulations (Rotello et al., 2008). To reiterate, here our goal is to find a valid measure to recognition memory, thereby eradicating the threat of replicating discoveries that are false.

**Why are false discoveries so prevalent in recognition memory?** Consider two types of models: the “data-generating model” and the “measurement model.” *We argue that for all standard SPSMs, there is good evidence that these two models are incongruent. Importantly, our simulations demonstrate that model incongruity gives rise to a high prevalence of false discoveries. In contrast, we claim that for d*_*a*_*, the two models are congruent and that the rates of false alarms are, in turn, curtailed at 5%.*

### Data-generating model

An often-overlooked fact is that empirical data do not come to the world in a haphazard way. Rather, data are sampled from a population with some form. The form may be a Gaussian, uniform, or any other theoretical distribution. Whatever the distribution, it has certain parameters (e.g., equal or unequal variances for targets and lures). In the best-case scenario, heated debates may exist regarding the population characteristics (e.g., Mickes et al., 2007; Rouder et al., 2010; Wixted & Mickes, 2010a). In the worst-case scenario, researchers are not even aware that the data are generated from a distribution. Irrespective of the epistemological knowledge concerning the population characteristics, the data are always sampled from a specific population. The term “*data-generating model*” refers to the population characteristics from which the data are sampled.

Three major families of data-generating models have been proposed to describe recognition memory. Here we only describe the best-known exemplars of each of these families. One family comprises continuous models, describing memory as mnemonic signals with different levels of strength (or familiarity; Hautus et al., 2022). The most popular exemplars of continuous models are the Gaussian signal detection models (equal-variance signal detection model; unequal-variance signal detection model or UVSD), which assume memory signals are generated from Gaussian lure and target distributions. The signals are subsequently compared to a decision criterion (representing their response bias) to generate an “old” (i.e., exceeding the criterion) or “new” (i.e., falling short of the criterion) response (Green & Swets, 1988).

A second family comprises threshold models, with the current leading model being the double-high threshold (2HT) model. Threshold models assume discrete memory states, in which an item is either detected as old or as new, or it is not detected at all and one is forced to guess (Swets, 1961; Snodgrass & Corwin, 1988). Compared to the Gaussian distributions assumed for the signal detection models, the 2HT model of recognition memory can be represented with rectangular distributions (Schütz & Bröder, 2011).

The third family, mixture models, suggest that a threshold process and a continuous process combine to produce recognition-memory signals. The leading representative of this family of models is the dual-process signal detection (DPSD) model (Yonelinas, 1994, 1999). The DPSD model assumes two components. The first component is “Recollection.” Recollection is assumed to be an “all-or-none” retrieval process unaffected by changes in criterion. According to the DPSD model, participants judge items as “old” if they find a recollection signal (i.e., detect that the item is old). If they do not detect the item to be old, the familiarity process comes into play.

The second component, the familiarity process, is described by Gaussian equal variance lure and target distributions. Participants respond “old” to items that produce familiarity signals that are higher than their criterion (again, representing their response bias). Items for which “old” responses are made constitute hits if they were sampled from the target distribution and are false alarms when sampled from the lure distribution. Thus, participants’ responses change as a result of changes in criterion. This familiarity component of the DPSD model is the signal detection processes, and it resembles the decision process assumed under the signal detection models described above (Yonelinas, 1994, 1999).

In pursuit of our goal of exploring each sensitivity measure’s validity under different data-generating models, we tested the measures under four data-generating models from the three families described above. Specifically, we sampled signals from distributions assumed by equal-variance signal detection, UVSD, 2HT, and DPSD models.

### Measurement model

All the measures that have been proposed to index performance in single-interval tasks are formulated based on unique assumptions regarding the nature of the (likely unknown) data-generating model^7^ (but see Footnote 4). The term “*measurement model*” refers to the set of assumptions upon which the measure is premised. Importantly, these assumptions imply a particular relationship between HR and FAR that can be expressed in a formula^8^ (i.e., HR equals some function of FAR). Note that inference from the measure (i.e., formula) to its data structure is bilateral. Given a measure, it is possible to infer the data structure it entails, and given a data structure, it is possible to derive a measure (Swets, 1986a, 1986b).

#### Implied and empirical ROC curves

The association between HR and FAR—their data structure—can be visually depicted using the receiver operating characteristic (ROC) curve, with HR on the ordinate and FAR on the abscissa (Hautus et al., 2022). Sensitivity is depicted as the distance from the major diagonal (representing chance performance, that is, HR = FAR). An ROC curve graphically illustrates the same level of sensitivity across all possible levels of bias. Therefore, all the operating points—[FAR, HR] pairs—that fall on the same ROC curve have identical sensitivity and differ only in bias (Hautus et al., 2022). The literature includes several different proposals about the true shape of the ROC curve in recognition memory (e.g., Wixted, 2007; Yonelinas & Parks, 2007; Rotello, 2017; Malejka & Broder, 2019).

The prevalence of false discoveries in recognition memory research is best understood when distinguishing between two types of ROC curves. The first is the *implied ROC curve*. The shape and parameters (e.g., slope) of an implied ROC curve are derived from a theoretical data-generating model. Typically, the implied ROC curve is the association between HR and FAR, as it is algebraically defined by the formula for computing sensitivity of a specific measure. Thus, the implied ROC of any measure is grounded in the assumptions of its measurement model.

The second type is the *empirical ROC curve* (see “Multi-point sensitivity measures and ROCs”), which is derived from the data by extracting multiple operating points and drawing the shape of the ROC based on those points^9^. The empirical ROC is not premised on any assumptions and provides a veridical (within sampling error) reflection of the true relationship—the true data structure—between the observed HRs and FARs in that particular experiment.

To illustrate, consider the sensitivity measure *P*_r_ = HR − FAR. If sensitivity = HR – FAR, then for a given magnitude of sensitivity, *M*_e_ (i.e., magnitude of sensitivity in a specific experiment), HR = *M*_e_ + FAR. Thus, the implied (i.e., predicted or theoretical) ROC curve for *P*_r_ is a linear function with a slope of 1 that intercepts the *y*-axis at the value *M*_e_, the sensitivity for the specific experiment. The formula of *P*_r_ thus *implies* a very specific relationship between HRs and FARs that is based on the assumptions of *P*_r_’s measurement model. This relationship, then, is the implied ROC for *P*_r_.

Let us assume that the assumptions of a measurement model are correct—that is, they reflect the true data structure produced by the data-generating model. If this is the case, then any two empirically derived operating points that have identical sensitivity (i.e., are drawn from the same lure and target distributions) will lie on the same implied ROC curve (within statistical error). In our example, if the measurement model of *P*_r_ represents the true data-generating model, then two [FAR, HR] pairs drawn from two experimental conditions with identical sensitivity should fall on the same linear ROC, which will have a slope of 1 and an intercept equal to *M*_e_.

The rationale that two iso-sensitive conditions should yield operating points that fall on the same empirical ROC curve breaks down when the two experimental conditions are assessed against an “incorrect” implied ROC curve (equivalently, when performance in the two conditions is indexed with a measure that is premised on the wrong model). In this case, the operating points obtained from two experimental conditions with truly identical levels of sensitivity may readily fall on different (incorrect) implied ROC curves. This, in turn, would lead to an erroneous inference that the conditions differ in sensitivity. Thus, incorrect inferences regarding sensitivity are likely to be made if two operating points are assessed against ROC curves that are derived from a measurement model that does *not* reflect the true data-generating model. In other words, *the observation that the two points fall on different implied ROC curves would constitute putative evidence that the points represent conditions with different levels of sensitivity, even though in truth, the conditions are equal in sensitivity.*

**Fig. 2:**
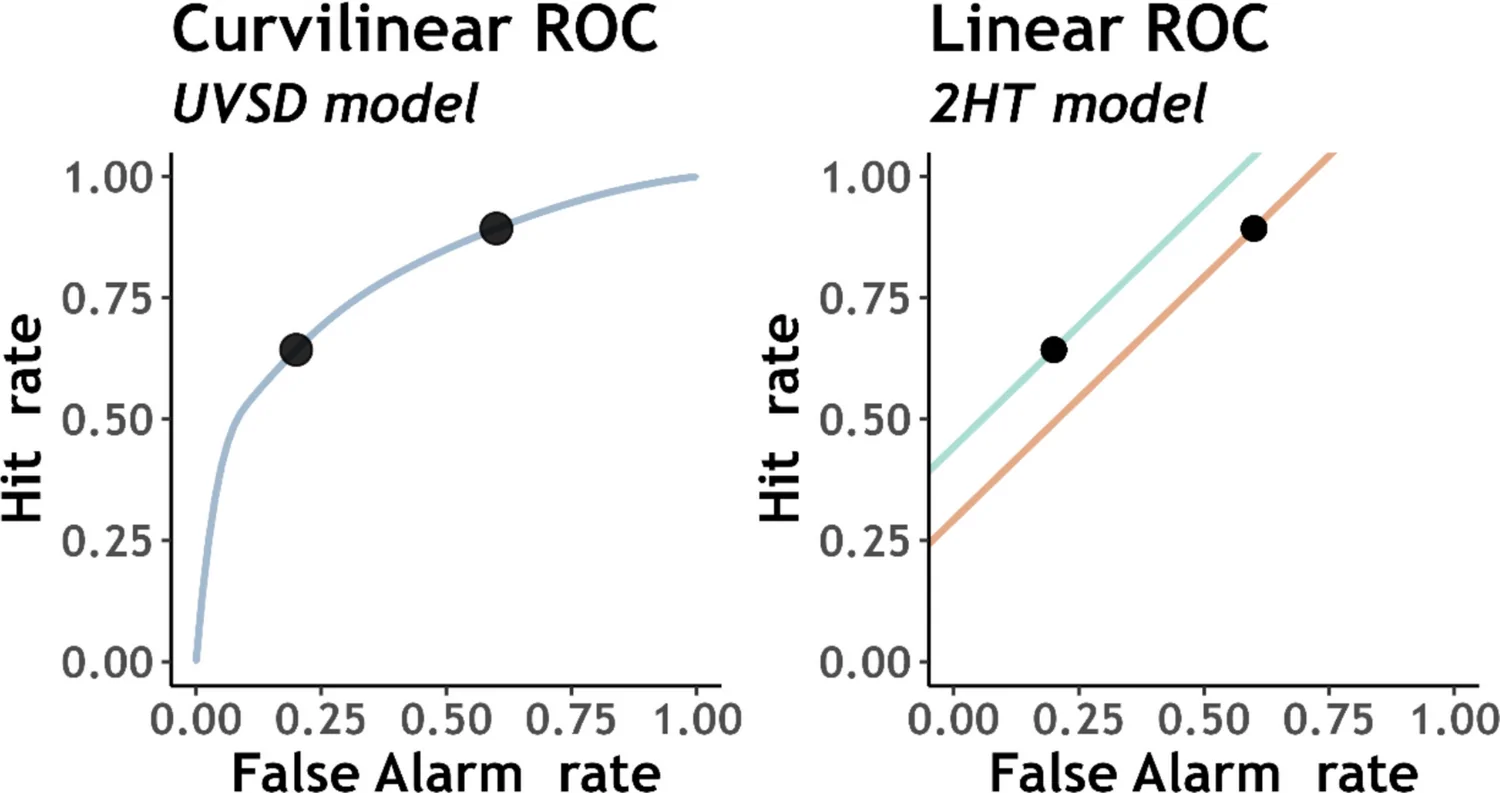
An example of two iso-sensitive operating points, obtained from Gaussian unequal-variance lure and target distributions. If the points are placed on a curvilinear ROC that is assumed by the unequal-variance signal detection model (left panel), they fit on the same curve. In contrast, the very same two points will fit on different curves when placed on a linear ROC that is assumed by threshold models

To illustrate, consider an implied ROC curve that is a linear function with a slope of 1 (e.g., the implied ROC of the sensitivity measure *P*_r_), but the observed (empirically obtained) operating points emerge from a different data-generating model, say, the unequal-variance Gaussian model (i.e., the UVSD model). In this case, it is likely that empirical operating points drawn from two experimental conditions with equal sensitivity will fall on two different implied (linear) ROC curves (see Fig. 2, right panel), leading to the erroneous conclusion that the conditions differ in sensitivity.

Critically, to incorrectly infer that operating points fall on different ROC curves—and to thus erroneously infer that an experimental manipulation affects sensitivity—it is not necessary to actually generate an implied ROC curve*.* Because different sensitivity measures represent different implied associations between HR and FAR, the use of a specific measure for indexing sensitivity in each condition is tantamount to fitting different operating points to an ROC curve. Thus, if a measure is based on the wrong data-generating model, two iso-sensitive conditions that differ in bias may erroneously be found to have different magnitudes of sensitivity. In conclusion, *discrepancies between the data-generating model and the measurement model are the root of the measurement crisis in recognition memory research*.

Once it has been established that incongruity between measurement and data-generating models can lead to erroneous inferences, simulation data are required to inform us regarding the *extent* of the problem. For example, the implied ROC curves of the DPSD model will often resemble an ROC curve derived from the UVSD model, and the difference between their ROC curves is not easily detected for some parameters of these models (see Fig. 6 for an illustration of the implied ROC curves under all the models we simulated). Therefore, while empirically derived operating points emanating from a UVSD data-generating model may fall on two different ROC curves implied by the DPSD model, it may also be difficult to establish that the distance between them is not a product of sampling error. To use more formal nomenclature, it is possible that some common measures of sensitivity are robust to incongruities between the measurement and data-generating model. By running simulations, we can estimate the extent of the problem of incongruity between the measurement and data-generating models. Although the Rotello et al. (2008) simulations failed to identify any common measures of sensitivity that are robust to incongruities between their measurement model and the data-generating model, those simulations did not include the DPSD model as a data-generating model, nor did they include *d*_a_ or *AUC*_g_ as measures of sensitivity.

A jaw-dropping finding reported by Rotello et al. was that when the data-generating and measurement models were incongruent, as sample size and number of trials increased, the Type I error rates (T1ER) increased to 100%^10^. Ponder this: *a 100% T1ER translates to results that will always replicate, despite being false*. As such, common sensitivity measures have high psychometric reliability, often yielding identical results across replications. However, these very same measures have low psychometric validity, in that they often do not index changes in sensitivity. Alas, the false scientific discoveries made with these measures cannot be disconfirmed by attempting to replicate them, even with longer experiments (more trials per participant) or with larger sample sizes. The experiments will replicate effortlessly.

Importantly, for a given data-generating model, there is no guarantee that a measure exists whose measurement model is congruent with that data-generating model. If such measure exists, all other measures that rely on other measurement models are categorically incongruent (Brady et al., 2022). Unfortunately, the research community has not converged on one consensual measure (see Fig. 1). This non-convergence is consistent with the fact that researchers differ in their belief of the veridical data-generating model (e.g., Wixted, 2007; Parks & Yonelinas, 2007; Mickes et al., 2007; Bröder & Schütz, 2009; Dube & Rotello, 2012; Rouder et al., 2010; Wixted & Mickes, 2010a). More pessimistically, the non-convergence might reflect lack of knowledge that each measure is premised on only one measurement model.

For two reasons, we propose *d*_a_ as a bias-independent measure of sensitivity. First, the measurement model of *d*_a_ is congruent with what is arguably the data-generating model of recognition memory—the UVSD model (unequal-variance Gaussian lure and target distributions; see below). Second, our version of *d*_a_ requires collecting multiple operating points from each participant so as to estimate the difference in variance for targets and lures, and to calculate the ensuing level of sensitivity. This approach entails collecting more data per participant and relying less on the assumptions of the measurement model (see below for the assumptions of the measurement model of *d*_a_). We now turn to describe different single and multiple point measures of sensitivity; after describing all measures, we will turn to our simulations.

### Single-point sensitivity measures

The common approach to measuring sensitivity relies on *a single* [FAR, HR] pair, therefore only requiring participants’ binary old/new judgments. When relying on a single operating point, a measure necessarily requires assumptions regarding the lure and target distributions—the aforementioned measurement model—to index a level of sensitivity for the data. Three of the most commonly used SPSMs are *P*_r_ (or, equivalently, *Percent* *correct*), *A*′, and *d*′. These three measures are based on three different measurement models.

#### *P*_r_ = HR – *FAR*

*P*_r_ is also known as “corrected hit probability” or “corrected hit rate” because the HR is “corrected for guessing” by subtracting the FAR from it. This measure is premised on threshold models (Hautus et al., 2022), in which the mnemonic information is assumed to be in a discrete, binary state (but see Rotello et al., 2006; Pazzaglia et al., 2013; Starns et al., 2014). Importantly, previous simulations (Rotello et al, 2008) tested *Percent* *correct* (*PC*), which equals. 5(HR + [1 − FAR]) =.5(HR − FAR) +.5 for experiments with an equal number of target and lure trials. *PC* is a linear transformation of *P*_r_ and is based on the same measurement model; therefore, the results for *P*_r_ and *PC* are interchangeable. In the current simulations, we tested *P*_r_ instead of *PC* because both *P*_r_ and *PC* are regularly used by researchers in the field (see Fig. 1). Rotello et al. demonstrated the low validity of *PC* in their 2008 simulations. Therefore, we chose to replicate the results with *P*_r_ in the current simulations. Demonstrating the low validity of both measures can help establish a better standard of measurement with a larger audience of researchers.

#### *A*′

Initially introduced as an “assumption-free” or “non-parametric” measure (Pollack & Norman, 1964), *A*′ was proven to implicitly assume a measurement model consistent with either rectangular or logistic distributions, depending on the magnitude of sensitivity (Macmillan & Creelman, 2004).

1 $${A}^{^{\prime}}=\left\{\begin{array}{c}\frac{1}{2}+\frac{\left(H-FA\right)\bullet \left(1+H-FA\right)}{4H\left(1-FA\right)},FA\le H\\ \frac{1}{2}+\frac{(FA-H)\bullet (1+FA-H)}{4FA(1-H)}, FA\ge H\end{array}\right.$$

#### *d*′

*d*′ is based on a Gaussian signal detection model. It measures the distance between the means of the lure and target distributions in units of their shared standard deviation. The underlying assumptions of *d*′ are based on the equal-variance signal detection model, assuming the lure and target distributions are equal-variance Gaussians (Tanner & Swets, 1954).

### Multi-point sensitivity measures and ROCs

While often overlooked, researchers can use multiple [FAR, HR] points to index sensitivity. The most common method for obtaining multiple operating points is based on variations in response bias (Green & Swets, 1988), created from confidence ratings. To obtain a confidence ROC (Hautus et al., 2008), judgments are made on a confidence scale (e.g., on a scale ranging from 1 to 6, 1 = highly confident new; 6 = highly confident old). Given confidence ratings, it is possible to create different bias cutoffs computed for each participant across all responses (Levi et al., 2021).

2 $${d}^{\prime}= z\left(HR\right)-z\left(FAR\right)$$

In our example of a six-point confidence scale, the most liberal cutoff is created by regarding as “new” responses of 1 and comparing them to responses of 2–6, regarded as “old.” HR and FAR can thus be computed for this scheme. A slightly less liberal cutoff scheme would be represented by tallying responses of 1 and 2, regarding them as “new,” and pitting them against responses of 3–6 viewed as “old.” This procedure continues until responses of 1–5 are regarded as “new” and compared to a response of 6, for a total of five [HR, FAR] pairs, that is, five operating points (for further explanation, see Mickes, 2015, or Hautus et al., 2022). When computed from participants’ confidence levels, each operating point reflects the same sensitivity level but a different level of bias (Hautus et al., 2022). The ROC curve can be transformed into *normal–normal*, or *Z* space (e.g., if H =.95, then *z*H = 1.645), yielding a *z*ROC curve. Because the *z*ROC is simply a transformation of the ROC, its features are also determined by the measurement model.

#### Mathematical modeling

Multiple operating points afford researchers with several potential approaches to estimating sensitivity. One such route is to fit the data to a specific mathematical model and then extract the model parameters, such as the estimates of the populations means and standard variations. Mathematical modeling can thus serve as a measurement tool (e.g., Greene & Naveh-Benjamin, 2022; Ford & Nieznański, 2024)^11^. For two reasons, we do not endorse this route.

First, measuring sensitivity via modeling is more challenging than it appears at first glance. In a seminal collaboration, Starns and his colleagues (2019) presented recognition memory experts with blinded data from seven different two-condition recognition experiments in which sensitivity, bias, both, or neither had been manipulated between conditions. The task for researchers was to infer what, if any, manipulation(s) had been imposed for each experiment. Across all data sets, experts’ conclusions were highly inconsistent, suggesting that modeling may not offer a solution to the memory crisis.

Second, our goal was to provide a solution to the measurement crisis for the entire research community, not only those equipped with the know-how to conduct mathematical modeling. We seek to grant all researchers a simple procedure, an algorithm, the result of which is a single scalar value per condition. Importantly, model fitting requires researchers to espouse a specified model (or several models). To illustrate, in our search of Springer Nature journals (Fig. 1), we found 33 articles that used model fitting as a means of estimating sensitivity in recognition data. Indeed, by fitting a model to the data, those researchers had no recourse but to assume a specific data-generating model. Remarkably, the models of choice in these studies were almost invariably threshold models^12^. The choice of these models is not trivial, given the evidence that threshold models are inferior to continuous models in fitting recognition memory data (Dubé et al., 2012; Pazzaglia et al., 2013; see Appendix B for details).

At bottom, mathematical modeling is not free of assumptions, just like the use of traditional measures such as *d*_a_. Ultimately, the traditional-measure approach is appealing because of its simplicity and accessibility. It is this route that we seek to advance in the current paper, through our investigation of *d*_a_.

#### Using an assumption-free measure (*AUC*_g_)

**Fig. 3:**
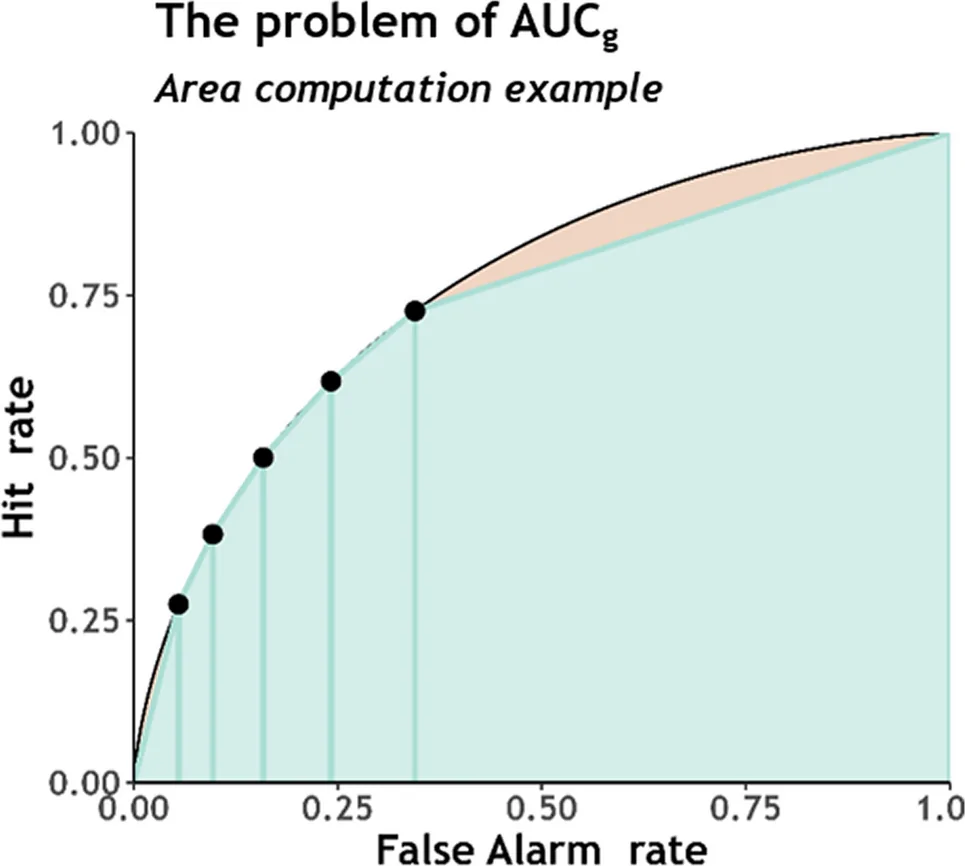
An example of the computation of the geometric area under the ROC curve (*AUC*_g_). The points in the graph represent possible operating points of participants, based on conservative criteria (our simulated conservative narrow criteria placement, setting C3 at 0.5 SDs relative to the lure distribution). The teal triangle and trapezoids represent the areas that are aggregated to compute this measure. The orange area represents the area that is omitted due to the computational requirement of extracting trapezoids. The size of the orange area is dependent on the location of the operating points

A different route to obtain bias-independent measures of sensitivity is to use an assumption-free measure (Hautus et al., 2022). The prime candidate is the geometric area under the ROC curve, labeled *AUC*_g_ (often denoted *A*_g_). *AUC*_g_ is a multi-point measure, computed by connecting successive [FAR, HR] points and adding vertical lines, parallel to the *y*-axis, from each operating point to the *x*-axis. This yields one triangle and several trapezoids (see Fig. 3). Aggregating the areas of the triangle and trapezoids obtains the value of *AUC*_g_ for the given ROC (Hautus et al., 2022). However, this is an approximation; the true area is the area under the continous ROC curve, not under the fragmented ROC curve plotted by connecting adjacent points with line segments. The price of the approximation are regions that are omitted from the calculation (orange areas in Fig. 3)^13^. Because the omitted area is dependent on the location of the operating points, its size changed as a function of the magnitude of bias. Our simulations will examine *AUC*_g_ to explore the extent to which the effects of bias impact the measurement of sensitivity. To anticipate the results, under many experimental scenarios, *AUC*_g_ was not bias-independent.

#### *d*_a_ and the UVSD model

Turning to our primary goal, investigating the properties of *d*_a_, specifically, as a multi-point measure. *d*_a_ is premised on only one parametric assumption: the lure and target distributions are Gaussian (consistent with the UVSD model). No assumption is made regarding the variance of the distributions. By suggesting *d*_a_ as a valid alternative to sensitivity measurement, we explicitly place our bets on the UVSD data-generating model. Our choice of model is based on our reading of the literature, which we summarize below. We emphasize that we do not wish to contribute to the literature on the nature of the data-generating model underlying recognition memory. Rather, our only goal is to address the measurement crisis and promote *d*_a_ as a bias-independent scalar measure of memory.

To infer the data-generating model of recognition memory, an important tool is the *z*ROC curve (see above, *“*Multi-point sensitivity measures and ROCs,” for explanation; Swets, 1986a; Wixted, 2007; Wixted & Mickes, 2010a, 2010b; Dubé & Rotello, 2023). If the underlying signal distributions are Gaussian, the *z*ROC curve is linear. In contrast, the 2HT model does not predict a linear *z*ROC (Hautus et al., 2022). For recognition data, the *z*ROC is indeed linear (Wixted, 2007; for a review see Wixted 2020).

A curvilinear ROC and a linear *z*ROC do not provide evidence that the underlying distributions are indeed Gaussian, however. The dual-process signal detection model (DPSD; Yonelinas, 1994) also predicts a curvilinear ROC and a (close to) linear *z*ROC (Yonelinas, 1999; Wixted, 2007). When directly compared against one another using group-level and individual-level ROCs, the UVSD model provides an overall better fit for the data (Heathcote 2003; Wixted 2007) compared to both the 2HT model and the DPSD model. Therefore, according to our reading of the literature, the best data-generating model for the recognition memory task is the UVSD model. Yet evidence in support of the competing models (2HT, DPSD) have also been reported (e.g., Malejka & Broder, 2019).

Under the UVSD model, the slope of the *z*ROC curve—labeled *S*—is an estimate of the lure-to-target ratio of the standard deviations (SDs). Empirical studies have consistently found that for recognition memory, *S* = 0.8 on average. This *S* value represents a target distribution that is 25% wider than the lure distribution (Lockhart & Murdock, 1970; Ratcliff et al., 1992; Dougal & Rotello, 2007; Mickes et al., 2007; Wixted & Mickes, 2010a).

If the UVSD model best describes the data-generating model of recognition memory, it implies that *all* the SPSMs are assumption-incongruent with the true data-generating model. *P*_r_ and *A*′ assume distributions that are not Gaussian. As for *d*′, it is indeed premised on a Gaussian distribution. However, *d*′ assumes the distributions to have equal variance, which is without doubt an indefensible assumption. Specifically, if the distributions underlying recognition memory are indeed Gaussian, multiple studies have shown that they are without doubt not of equal variance (Ratcliff et al., 1992; Dougal & Rotello, 2007; Mickes et al., 2007). Stated differently, conditional on the distributions being Gaussian (the measurement model of *d*′), they are not equal variance—an assumption of the measurement model of *d*′. Thus, the measurement model of *d*′ is indisputably incongruent with the data-generating model of recognition memory, consistently reflected by a *z*ROC slope of around 0.8.

We propose that *d*_a_ may be the best alternative for measuring sensitivity in recognition memory because it has a measurement model that is congruent with that of the UVSD data-generating model. Specifically, we suggest that using *d*_a_ as a multi-point measure can account for any differences in lure-to-target SD ratios (henceforth, SD ratios) between experiments or participants (see below), therefore fitting the UVSD data-generating model best. We now turn to describe this measure and our multi-point version.

Like *d*′, *d*_a_ is based on signal detection theory and entails the subtraction of *Z*-transformations of HR and FAR. Unlike *d*′, *d*_a_ is not premised on an equal-variance assumption, as we explained above. *d*_a_ is defined as the distance between the means of the lure and target distributions, measured in the root-mean-square average of their SDs (Swets & Pickett, 1982). *d*_a_ is defined as

3 $${{\boldsymbol{d}}}_{{\boldsymbol{a}}}=\sqrt{\frac{2}{1+{{\boldsymbol{s}}}^{2}}}\bullet\left({\boldsymbol{z}}\left({\boldsymbol{H}}{\boldsymbol{R}}\right)-{\boldsymbol{s}}\bullet {\boldsymbol{z}}\left({\boldsymbol{F}}{\boldsymbol{A}}{\boldsymbol{R}}\right)\right)$$

The measurement model of *d*_a_ can support the UVSD^14^ data-generating model. Importantly, *d*_a_ is incongruent with the 2HT model (the measurement model of *P*_r_ and PC) and is likewise incongruent with the assumptions of the DPSD model, specifically the nonlinearity of the *z*ROC slope, despite the resemblance in ROC predictions (Wixted, 2007). By substituting the lure-to-target SD ratio (see Eq. 3) with the *z*ROC slope, *S*, we get a unique (multi-point) way to use *d*_a_ as a measure.

Researchers might (incorrectly) consider *d*_a_ as a valid measure of recognition memory when inserting a fixed value of 0.8 as the SD ratio. However, substituting a mean value previously found in experiments for the SD ratio in Eq. 3 does not take into consideration the variability in the magnitude of this ratio, and of *S*, across participants. It turns out that the value of *S* varies across participants, with values as low as 0.5 for some participants and higher than 1 for others (Ratcliff et al., 1992; Macmillan et al., 2004). In Appendix C, we show that using 0.8 as the SD ratio produces a valid measure only under very specific scenarios where the data-generating model is unequal-variance Gaussian distributions with the SD ratio of exactly 0.8 (all appendices are available through the OSF repository: https://osf.io/d5jub).

Our algorithm for computing *d*_a_ explicitly takes this SD ratio variability into account. We estimated *S* individually for each participant based on the operating points of their empirical *z*ROC curves created from six criteria that were placed across the lure and target distributions, yielding five operating points (see Method). For more accurate estimates of the true SD ratio, for each participant, we computed *S* separately in each condition and then used the mean of the two *S* values for the estimation of *d*_a_ in both conditions (but see Appendix C for an evaluation of alternative ways of computing *S*). By using this algorithm, only one assumption was made about the data-generating model, that of Gaussian lure and target distributions. We eschewed the indefensible equal-variance assumption.

Simulations are a particularly apt tool for our investigation, as they offer the external criterion for validation that is missing in empirical research. Although they do not provide proof that *d*_a_ is a valid measure of sensitivity in empirical setting, they do provide a comprehensive assessment of the performance of *d*_a_ (and the other tested measures) under iso-sensitive conditions. Importantly, the simulations show the boundary conditions of *d*_a_—if and under what circumstances the Type I error rate exceeds 5%, even if the simulated conditions are iso-sensitive.

Our simulations of *d*_a_ were run to achieve two goals. First, simulations allowed us to test whether *S* can be used to estimate the SD ratio individually for each participant under optimal conditions with minimal noise. Our choice of algorithm for estimating *S* was not trivial, and simulations were required to support its feasibility (see Appendix C). Second, simulations can identify what assumptions are required for *d*_a_ to be considered valid, specifically the various assumptions of the different data-generating models. We tested whether *d*_a_ is bias-independent under all three families of models described above: the 2HT model, DPSD model, and, most importantly, the UVSD model.

For all measures, we used the same lure and target distributions for both conditions and created a difference in bias by setting Condition 1 to have more liberal criteria (the *liberal condition*) and Condition 2 to have more conservative criteria (the *conservative condition*). If a measure of sensitivity is valid, we should obtain the same index of sensitivity for both conditions, reflected in a T1ER of approximately 5%.

We predicted two main results. First, common SPSMs (*P*_r_, *A*′, and *d*′), as well as *AUC*_g_, will be found to be bias-dependent and will yield high rates of Type I errors that will increase with sample size and number of trials. Second, *d*_a_ will be found to be a bias-independent measure of sensitivity when Gaussian distributions will be assumed (but not under the 2HT model). For data generated from the DPSD model, our prediction was more nuanced. The implied ROC curves of the DPSD model and the UVSD model are often hard to distinguish (see Fig. 6, middle and right panels). Therefore, we predicted *d*_a_ will show approximately 5% T1ERs under different DPSD model’s simulations. However, in boundary conditions, when the ROC that is obtained from the DPSD model diverges from that of the UVSD model (see Fig. 6), we predicted *d*_a_ will no longer be bias-independent.

## Method

We ran 10,000 iterations for each of 1,962 Monte Carlo simulations with the goal of testing the validity of different sensitivity measures. We simulated experiments of two-level (i.e., two conditions) within-participant designs. The two conditions were iso-sensitive, that is, the lure and target distributions (and thus, the distance between their means) were the same for both conditions. In other words, the simulated data-generating model was identical for both conditions, except for the value of the parameter that determines response bias in that model.

Sensitivity was indexed with five measures: three SPSMs (*P*_r_, *A*′, and *d*′) and two multi-point measures (*AUC*_g_ and *d*_a_). Each of the five measures was tested against distributions consistent with each of four data-generating models: the 2HT model, the DPSD model, the equal-variance signal detection model, and, most importantly, the UVSD model. Each model entailed different distributional parameters (e.g., rectangular distributions for the 2HT model; unequal-variance Gaussian distributions for the UVSD model). To simulate our data, we randomly sampled values of signals from lure and target distributions, with each sampled value representing the memory strength in a particular trial. The values of means and SDs are listed below (see “Distribution (data generation) variables”).

Following Rotello et al. (2008), for each participant in a particular simulation, we used the same sampled signal values for the two iso-sensitive conditions. By choosing this approach, we matched the two sets of simulated trials, thereby simulating data from a within-participant design^15^.

While the conditions were created to be iso-sensitive, they were modeled to have different decision criteria against which the sampled signal strengths were evaluated (see “Criteria variables” for details). When sampled from the target distribution, signal strengths above a given criterion reflected a positive (“old”) response that when calculated across trials yielded a HR value. Similarly, the signals sampled from the lure distribution were compared to different decision criteria to compute participants’ false alarm rates across trials.

**Fig. 4:**
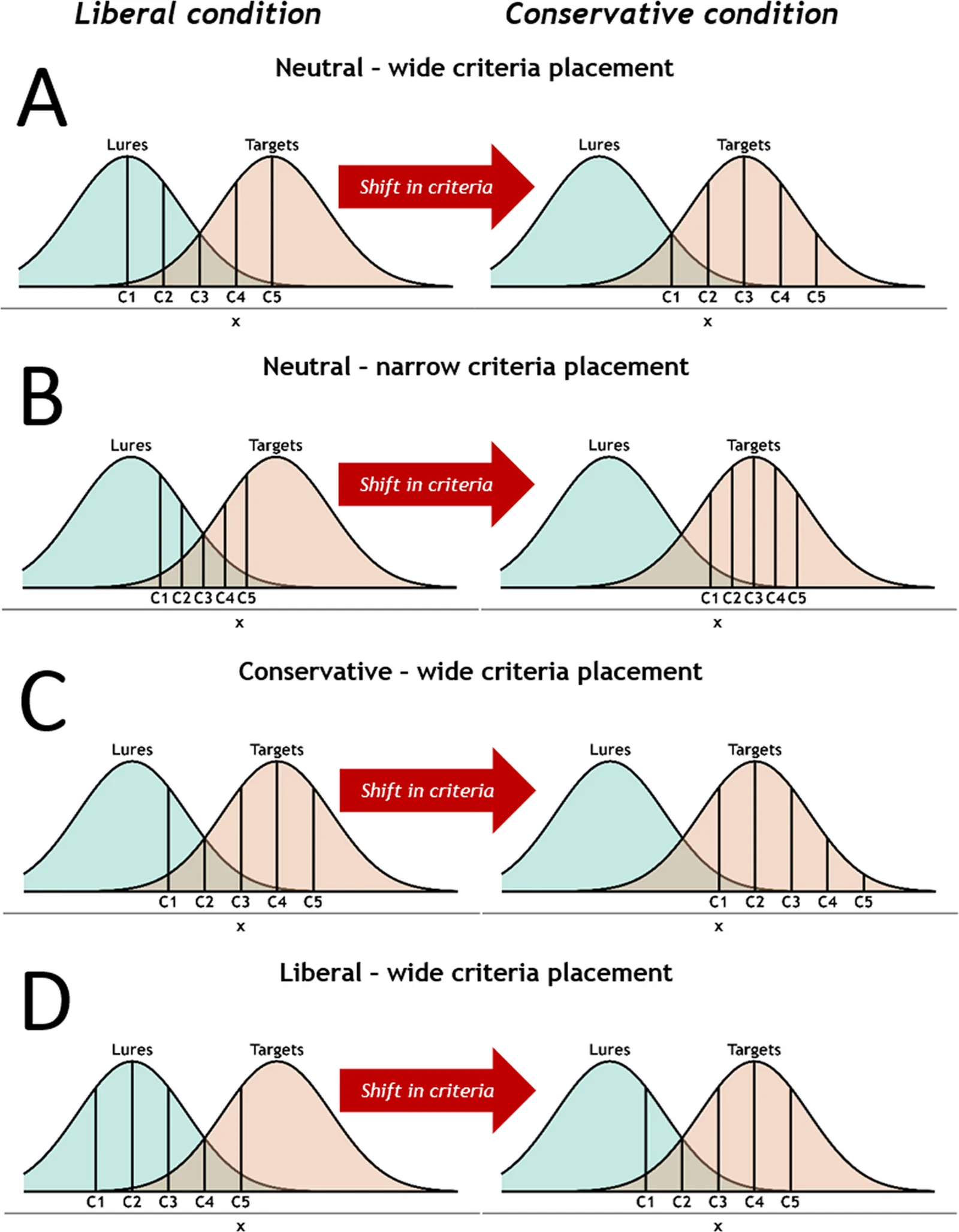
Illustration of different criteria placements in the simulations, and the shift between the liberal (left) and conservative (right) conditions for the different placements. The two top rows represent neutral criteria placement, in both wide (panel **A**) and narrow (panel **B**) versions. The bottom rows represent a conservative (panel **C**) and a liberal (panel **D**) placement. The illustrations depict cartoons of the criteria placement. They do not reflect the true values used in the simulations. The true values appear in the main text and in Fig. 5

**Fig. 5:**
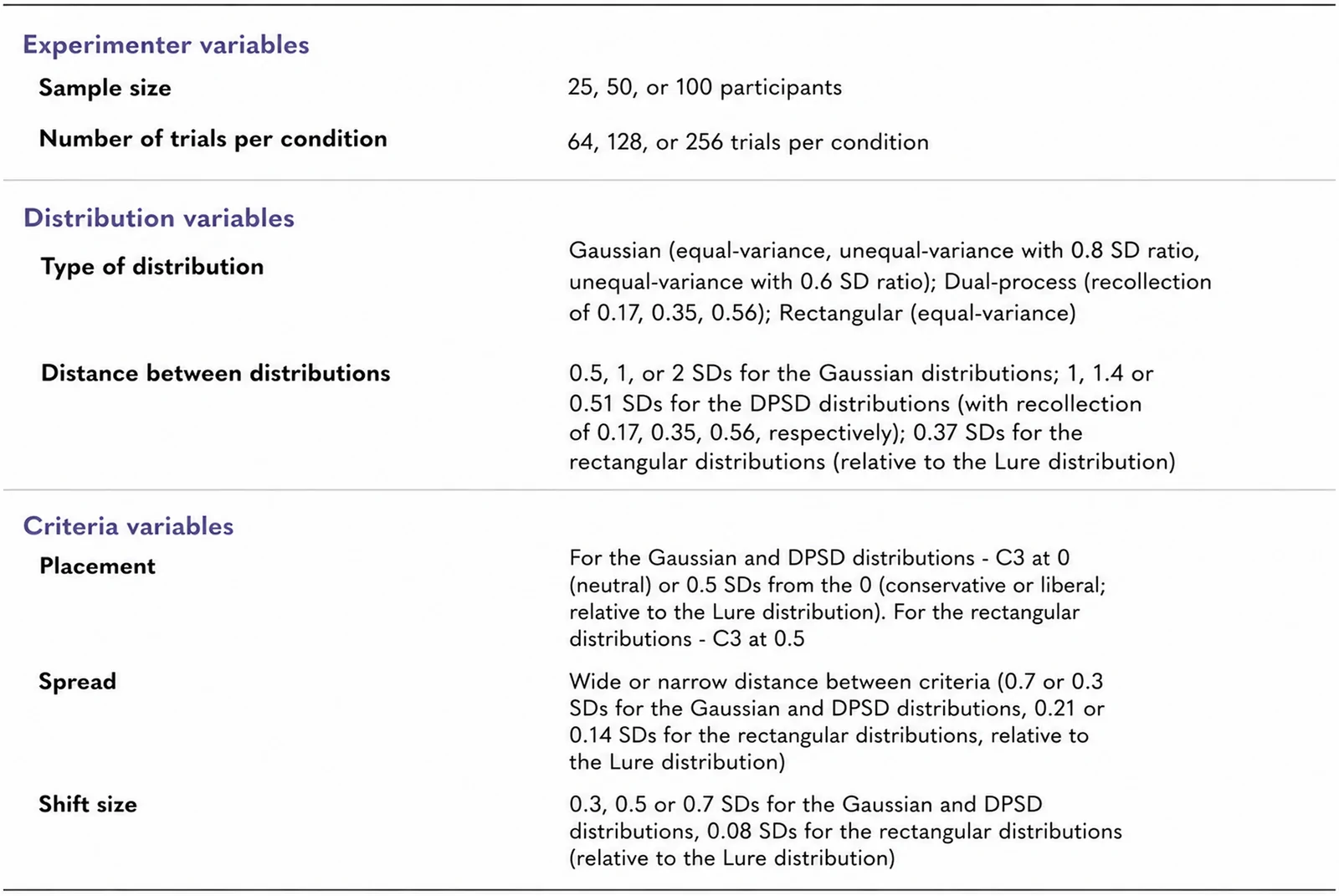
Summary of all the variables and their levels used in the simulations to explore the parameter space. All variables are categorized by variable types. The variable types appear in the left column. The variables (parameters) and their chosen values appear in the middle and right column, respectively

Five criteria were needed to simulate a six-point confidence scale, henceforth labeled C1–C5 (see Fig. 4 and theoretical explanation in the introduction, “Multi-point sensitivity measures and ROCs”). We simulated participants’ responses by comparing each signal to each of the five criteria (see Fig. 5 for the criterion values). When summed across all simulated trials, this yielded five [FAR, HR] operating points. Appendix D provides an illustration and an explanation of how different points are extracted from each of the criteria. Note that all appendices are available through the OSF repository: https://osf.io/d5jub.

Although *d*_a_ is derived from multi-point data, its final computation (see Eq. 3) uses only a single pair of HR and FAR—specifically, those defining the middle operating point of the ROC. In practice, using a confidence scale, this point is obtained by splitting the scale into two response categories, treating higher ratings (e.g., 4–6) as “old” responses and lower ratings (e.g., 1–3) as “new.” Thus, we used the HR and FAR values resulting from signals exceeding the middle criterion (C3, see Fig. 4). This is the same operating point used in the calculation of traditional SPSMs; thus, *d*_a_ and indices such as *P*_r_, *A*′, and *d*′ may be compared directly.

For the computation of *AUC*_*g*_, multiple opertating points were needed. As described in the introduction, we used the trapezoid method, where we connected the adjacent operating points, thus creating trapezoids and a triangle. We then computed the respective areas (see Introduction, “Multi-point sensitivity measures and ROCs”). Finaly, we summed the values of all the areas to obtain an approximation of the area under the ROC curve, *AUC*_g_ (see Fig. 3).

Likewise, for the calculation of *d*_a,_ specifically the value of *S,* multiple operating points were needed. The points were subsequently converted to *z*-space (see Appendix D for an explanation on how to convert operating points to *z*-space; e.g., if HR = 0.95, its value was converted to 1.645 in *z*-space). Using the *z*-scores of the operating points (i.e., the *z*ROC curve), we used linear regression to extract the slope (*S*) and to estimate the SD ratio individually for each simulated participant (using the mean *S* across conditions). Note that, because the transformation to *z*-scores is not possible for values of HR = 1 and FAR = 0, several simulated participants have fewer than five distinct operating points. Because the shape of the *z*ROC (i.e., curved, linear) cannot be accurately established with fewer than three operating points, our algorithm required a minimum of three operating points in each condition.

As described in detail in Appendix C, we tested four additional strategies for estimating the SD ratio: (1) the same as above but with a minimum of two (rather than three) operating points; (2) a constant value of *S* = 0.8 for each participant and condition (i.e., no estimation); (3) a single value of *S* for all participants and conditions, estimated from the mean *z*ROC slope across the entire sample and both conditions; and (4) the same two values of *S* for every participant (one for each condition), estimated from the mean *z*ROC slope across the entire sample in each condition. None of these alternative estimation strategies performed as well as the algorithm that required a minimum of three operating points in each of the two conditions. Specifically, each of the alternative strategies yielded higher T1ERs than the three-point, mean-of-two-conditions, individually estimated approach for at least some of the tested data-generating models (see Appendix C for details). Therefore, in our simulations, we used this three-point algorithm to estimate the SD ratio.

It should be noted that when using signal detection sensitivity measures, rates of 0 or 1 (i.e., 0% and 100%, respectively) cannot be converted to *z*-scores because they will result in a value of ∞ (or −∞). Possible solutions to this problem involve slightly adjusting rates of 0 to be very small positive numbers, and rates of 1 to be values close to 1. Specific correction methods (e.g., log-linear correction methods, Snodgrass & Corwin, 1988) are dependent on assuming a particular data-generating model. However, when analyzing the data of participants with a minimum of three operating points, C3 will invariably yield HR values lower than 100% and FAR values higher than 0%. At bottom, under our optimal algorithm that requires a minimum of three operating points per condition, the HR and FAR used for calculating sensitivity (see Eqs. 2 and 3) were never equal to 100% and 0%. Hence, a correction method is required neither for the calculation of *d*_a_ nor for the calculation of d′. Interested readers are referred to Rotello et al. (2008), who tested several correction methods for SPSMs in the context of T1ER simulations.

Ultimately, following Rotello et al. (2008), we sampled the parameter space with several experimental scenarios. These scenarios varied across three types of study variables, with their levels completely crossed. The variable types were *experimenter variables*—variables that are controlled by the experimenter in an empirical setting (sample size, number of trials); *distribution variables—*variables that describe the data-generating model, usually unknown to the experimenter; and *criteria variables*—the placement and arrangement of the different confidence levels, corresponding to participants’ possible levels of bias. See Fig. 5 for a summary of all the variables and their levels.

### Experimenter variables

Nine combinations were created by fully crossing three sample sizes (*N* = 25, 50, or 100) with three numbers of trials per condition (*T* = 64, 128, or 256).

### Distribution (data generation) variables

We simulated experimental scenarios based on four different data-generating models. The first three simulated models were *single-process* data-generating models. Under this category, we simulated two continuous models, the equal-variance signal detection and the UVSD model, both of which assume Gaussian lure and target distributions. We also simulated one discrete-state model, the 2HT model, which is consistent with underlying evidence distributions that are rectangular in form. To reiterate, the signals simulated from the distributions assumed under the single-process models were compared to the criteria we placed (i.e., C1–C5) to produce “old” or “new” responses.

The fourth simulated data-generating model was a *mixture model* (DPSD), which assumes that participants’ signals are generated from two different postulated processes, familiarity and recollection. Familiarity signals are assumed to generate from a continuous process. The familiarity-based distributions are equal-variance Gaussian lure and target distributions (Yonelinas, 1994, 1999). Participants’ criterion placement (i.e., their level of bias) is assumed to only impact the familiarity process, with signals higher than the criterion either constituting hits (when sampled from the target distribution) or false alarms (when sampled from the lure distribution). In contrast, recollection signals, the second postulated process, are assumed to arise from an all-or-none threshold process (representing a single-high threshold, or 1HT, model because only studied items can be recollected).

To simulate the data for the DPSD model, a fixed proportion of the target trials represented recollection. To achieve this, we set those simulated trials to have a signal strength of +∞, therefore exceeding any chosen criteria and reflecting the highest-confidence response. The selected recollection proportion was a distribution parameter, with different values across different simulations. The remaining trials represented familiarity. They were sampled from equal-variance Gaussian distributions with a target mean higher than that of the lure. The distance between those distributions was also a distribution parameter. The recollection (proportion of high-confidence recollection trials) and familiarity (distance between lure and target Gaussian distributions) parameters were based on typical values reported in the DPSD literature (see Yonelinas, 2002; Wixted, 2007) and are listed in Fig. 5.

For all data-generating models, we simulated scenarios of different distances, *d,* between the means of the lure and target distribution in units of SDs relative to the lure distribution. A larger distance, *d*, between the distributions reflects a higher sensitivity for distinguishing between targets and lures. For the Gaussian distributions, *d* = 0.5, 1, and 1.5 SDs relative to the mean of the lure distribution. For the rectangular distributions, *d* = 0.37 SDs relative to the mean of the lure distribution^16^. For the DPSD model, we set three pairs of recollection and familiarity parameters chosen based on the articles mentioned above: *recollection* = 0.17 and *d*_familiarity_ = 1 (Wixted, 2007); *recollection* = 0.35 and *d*_familiarity_ = 1.4 (Wixted, 2007); *recollection* = 0.56 and *d*_familiarity_ = 0.51 (Yonelinas, 2001).

**Fig. 6:**
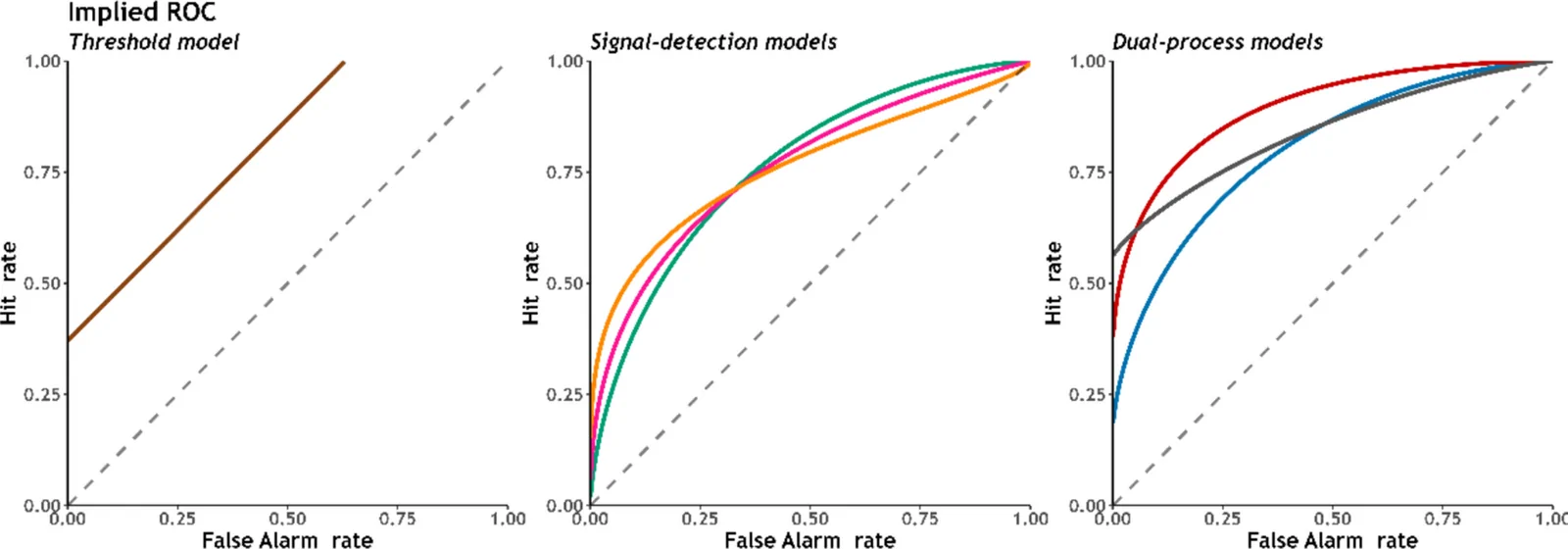
Illustration of implied ROC curves of the simulated data-generating models. Left panel: implied ROC of 2HT model (assuming rectangular distributions). Middle panel: implied ROCs of signal detection models (green curve: assuming equal variance Gaussian distributions; pink: unequal variance Gaussian distributions with SD ratio of 0.8; orange: unequal variance Gaussian distributions with SD ratio of 0.6). Right panel: implied ROCs of DPSD models (blue: assuming *recollection* = 0.17 and *d*_familiarity_ = 1; red: *recollection* = 0.35 and *d*_familiarity_ = 1.4; gray: *recollection* = 0.56 and *d*_familiarity_ = 0.51). The dashed line in each panel (i.e., major diagonal) represents chance-level performance

As explained in the introduction, each data-generating model entails different assumptions, thus yielding a different implied ROC curve. Figure 6 depicts the implied ROC curves of our simulated data-generating models. The left panel depicts an implied ROC based on threshold models, consistent with signals generated from equal-variance rectangular distributions. The middle panel depicts implied ROC curves of the equal-variance signal detection and the UVSD models, consistent with equal-variance (green curve) and unequal-variance (pink: SD ratio = 0.8, orange: SD ratio = 0.6) Gaussian distributions. The right panel depicts the implied ROC curves of the DPSD model using our simulated distribution variables (blue, *recollection* = 0.17 and *d*_familiarity_ = 1; red, *recollection* = 0.35 and *d*_familiarity_ = 1.4; gray, *recollection* = 0.56 and *d*_familiarity_ = 0.51). Examination of the middle and right panels reveals that DPSD-based implied ROC curves can be more (right panel, blue curve) or less (right panel, grey curve) similar to the UVSD implied ROC (middle panel, pink curve), conditional on the choice of the recollection and familiarity parameters.

### Criteria variables

We set criteria under different schemes for the liberal condition and defined the conservative condition relative to the liberal (see Fig. 4). For simplicity, the criteria were always equally spaced, with distances between criteria that were either *narrow* or *wide* as defined in Fig. 5. For the liberal condition, we manipulated the general locations of the criteria (liberal, neutral, or conservative) as well as their spread (wide or narrow).

For the conservative condition, we shifted each of the five liberal criteria by a fixed magnitude. We created different experimental scenarios by varying the magnitude of the shift, as described in Fig. 5. For measures that are affected by bias, a bigger shift in bias is expected to lead to more erroneous significant results (i.e., higher T1ER).

In summary, the range of parameter values we selected resulted in 1,962 combinations of assumptions about the data-generating model and the experimental design choices (e.g., sample size). For each of those 1,962 combinations, we ran 10,000 “experiments” using Monte Carlo simulations, and for each experiment, we ran a set of paired *t*-tests comparing the observed sensitivity across conditions for each of the five measures of sensitivity (*P*_r_, *A*′, *d*′, *AUC*_g_, and *d*_a_). Both the data and the simulations script are available through the OSF link: https://osf.io/d5jub.

## Results

We defined a simulation as a particular combination of parameters, with identical values across all the parameters (sample size, number of trials, type of distribution, distance and criteria placement). For each simulation, we calculated the observed HR and FAR in each condition for each participant with at least three operating points per condition. We then computed all the sensitivity measures of interest (*P*_r_, *A*′, *d*′, *AUC*_g,_ and *d*_a_). The sensitivity measures computed for each participant in the two conditions were then subject to a within-participant *t*-test with α =.05; each simulation was repeated 10,000 times. The dependent variable was the Type I error rate (T1ER), which was the observed rate of significant results across the 10,000 repetitions. The detailed results for each simulation appear in Appendix A at the OSF repository (https://osf.io/d5jub). Here we provide representative findings.

### Type I error rates as a function of sample size

**Fig. 7.**
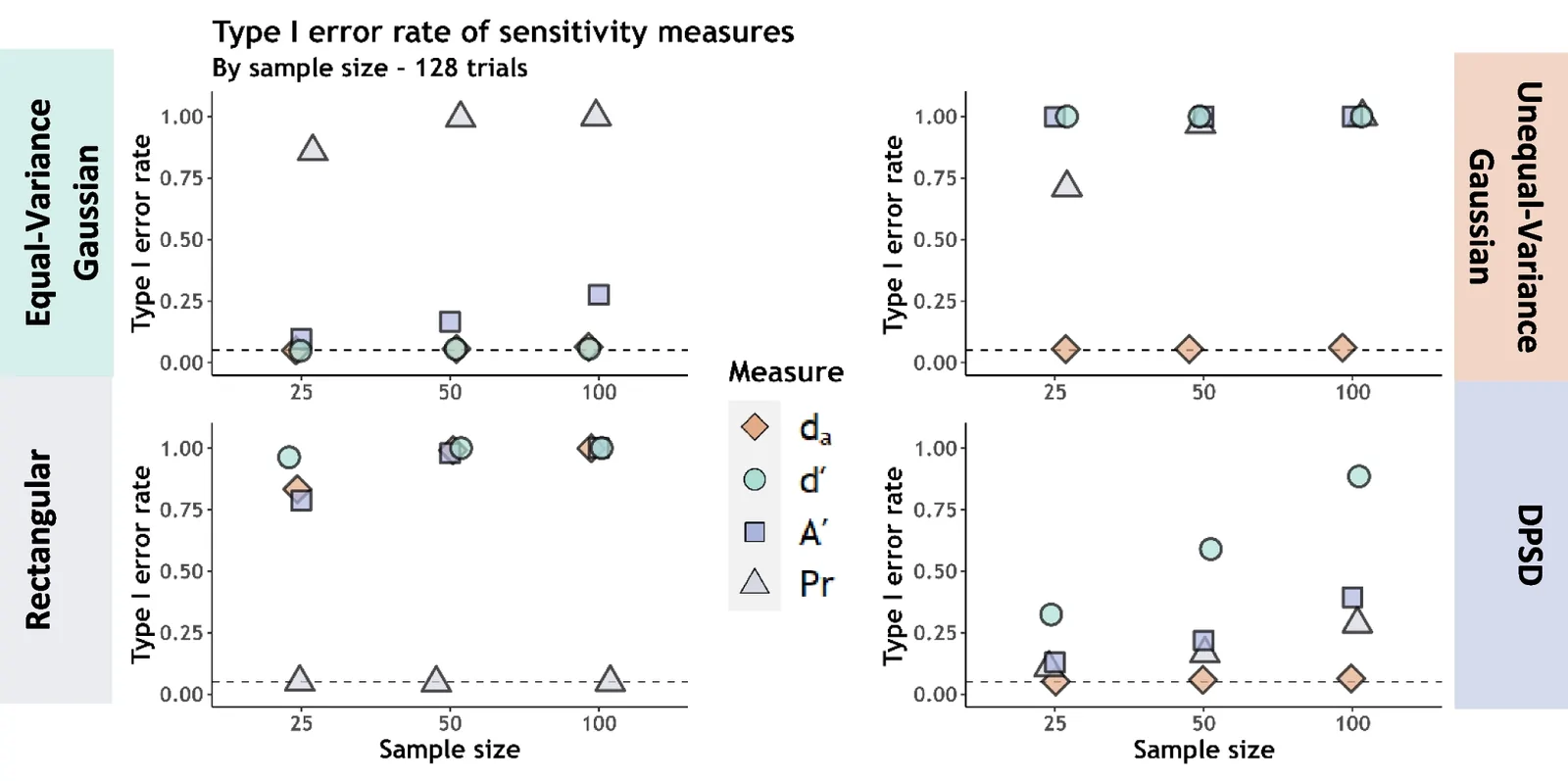
Type I error rates of *P*_r_ (triangles), *A*′ (squares), *d*′ (circles), and *d*_a_ (diamonds) as a function of sample size, under Gaussian (equal variance, top-left panel; unequal variance with SD ratio = 0.8, top-right panel), rectangular (bottom-left panel), or DPSD (bottom-right panel) distributions. Number of trials was simulated as 128 trials per condition. For Gaussian distributions, the spread of criteria was neutral and wide. The distance between distributions was 1 SD (relative to the lure distribution), and the size of shift in criteria between conditions was 0.5 SDs (relative to the lure distribution). For rectangular distributions, the spread of criteria was neutral and narrow. The distance between distributions was 0.37 SDs (relative to the lure distribution), and the size of shift in criteria between conditions was 0.08 SDs (relative to the lure distribution). For DPSD, the spread of criteria was also neutral and wide, and the size of shift in criteria between conditions was 0.5 SDs (relative to the lure distribution). The proportion of recollection was 0.17, and the distance between the familiarity target distribution and lure distribution was set at 1 SD (relative to the lure distribution). The dashed line indicates *α* =.05

Figure 7 presents T1ERs of the different measures of sensitivity as a function of sample size (25, 50, or 100 participants per experiment) and data-generating model.^17^

#### *d*_a_

It is quite apparent that *d*_a_ stands out from the crowd of sensitivity measures. The most important pattern depicted in Fig. 7 is that *d*_a_ was the only measure to display acceptable T1ERs of 5% for both equal and unequal Gaussian distributions across all values of sample size. Ultimately, by using *S* as an estimate of the lure-to-target SD ratio, *d*_a_ was able to index sensitivity independent of bias across Gaussian data-generating models with different ratios of lure-to-target SD and across different criteria placements. This finding was the same for all simulated sample sizes.

As expected, for rectangular distributions, T1ERs of *d*_a_ were typically higher than 5% (see Fig. 7, bottom-left panel), rising as sample size increased. This finding was predicted because the threshold data-generating model assumes rectangular distributions that are incongruent with the Gaussian assumption of the measurement model underlying *d*_a_.

Interestingly, for DPSD distributions, T1ERs of *d*_a_ were usually close to 5% (see Fig. 7, bottom-right panel). This finding is not surprising as it might seem at first glance. As we described in the introduction, the shape of the ROC curve implied by the DPSD model for the specific parameters depicted in Fig. 7 closely resemble those implied by the measurement model of *d*_a_ (i.e., UVSD model). Figure 6 depicts the implied ROC curves for both models (see the pink curve in the middle panel and the blue curve in the right panel), revealing only a minute discrepancy between their ROCs. Therefore, under the typical scenario depicted in the bottom-right panel of Fig. 7, *d*_a_ is a valid index of sensitivity and is not affected by bias, even when large sample sizes are simulated.

**Fig. 8:**
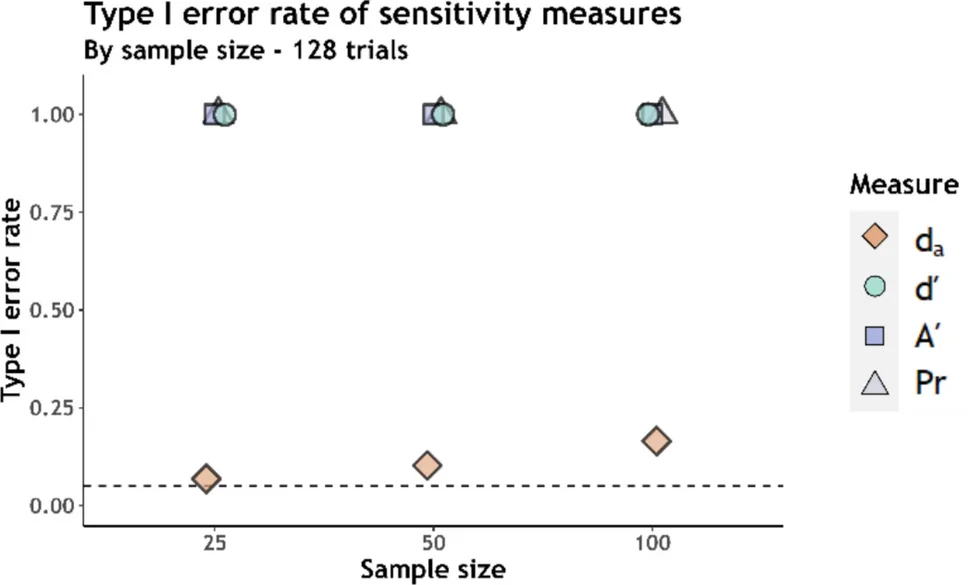
Type I error rates of *P*_r_ (triangles), *A*′ (squares), *d*′ (circles), and *d*_a_ (diamonds) as a function of sample size under the DPSD model. The number of trials was simulated as 128 trials per condition. The proportion of recollection was 0.56, and the distance between the familiarity target distribution and lure distribution was simulated as 0.51 SDs (relative to the lure distribution). The spread of criteria was neutral and wide, and the size of shift in criteria between conditions was 0.5 SDs (relative to the lure distribution). The dashed line indicates α =.05

To the extent that Type I errors might occur using *d*_a_ under the DPSD model, they would do so only under more extreme scenarios (e.g., large shift in criteria between conditions or a large contribution of recollection; see Appendix A). To illustrate this point, consider the shape of the orange curve in the right panel of Fig. 6, which depicts the implied ROC of the DPSD model assuming *recollection* = 0.56 and *d*_familiarity_ = 0.51 (these values were reported in modeling work; Yonelinas, 2001). This is the highest proportion of recollection trials that we simulated for DPSD, therefore the largest discrepancy between the data-generating model and measurement model’s implied ROCs. T1ERs of the SPSMs and *d*_a_ for these DPSD parameters are shown in Fig. 8. As is clear in Fig. 8, the large discrepancy between models resulted in T1ER for *d*_a_ that exceeded 5%, reaching about 16% for the largest simulated sample size. We address the T1ERs of *d*_a_ in DPSD simulations in the discussion.

#### SPSMs

Our findings regarding the measurement of sensitivity with the different SPSMs fully replicated those of the original simulations (Rotello et al., 2008). Specifically, across the parameter space, T1ERs for SPSMs were approximately 5% under only two conditions. First, for *d*′ under equal-variance Gaussian distributions (Appendix A). Second, for *P*_r_ under rectangular distributions (also see Appendix A). Notwithstanding these two conditions, for all other simulated experimental conditions, the T1ERs for the SPSMs were higher than 5% and rose to 100% as sample size increased.

We especially note the simulations of unequal-variance Gaussian distributions (likely the distributions underlying recognition memory) for which all SPSMs showed high T1ERs. The top-right panel of Fig. 7 depicts the T1ERs of *d*′, *A*′, and *P*_r_, all of which are well above the 5% dashed line, increasing up to approximately 100% as a function of sample size.

In summary, our results are consistent with the idea that T1ERs are approximately 5% if and only if the data-generating model is congruent with the measurement model (i.e., T1ER of *d*_a_ for all types of Gaussian distributions; T1ER of *d*′ for equal-variance Gaussian distributions; T1ER of *P*_r_ for rectangular distributions). Once the models are incongruent, T1ERs exceed 5% and increase as a function of sample size.

### The effect of number of trials

**Fig. 9:**
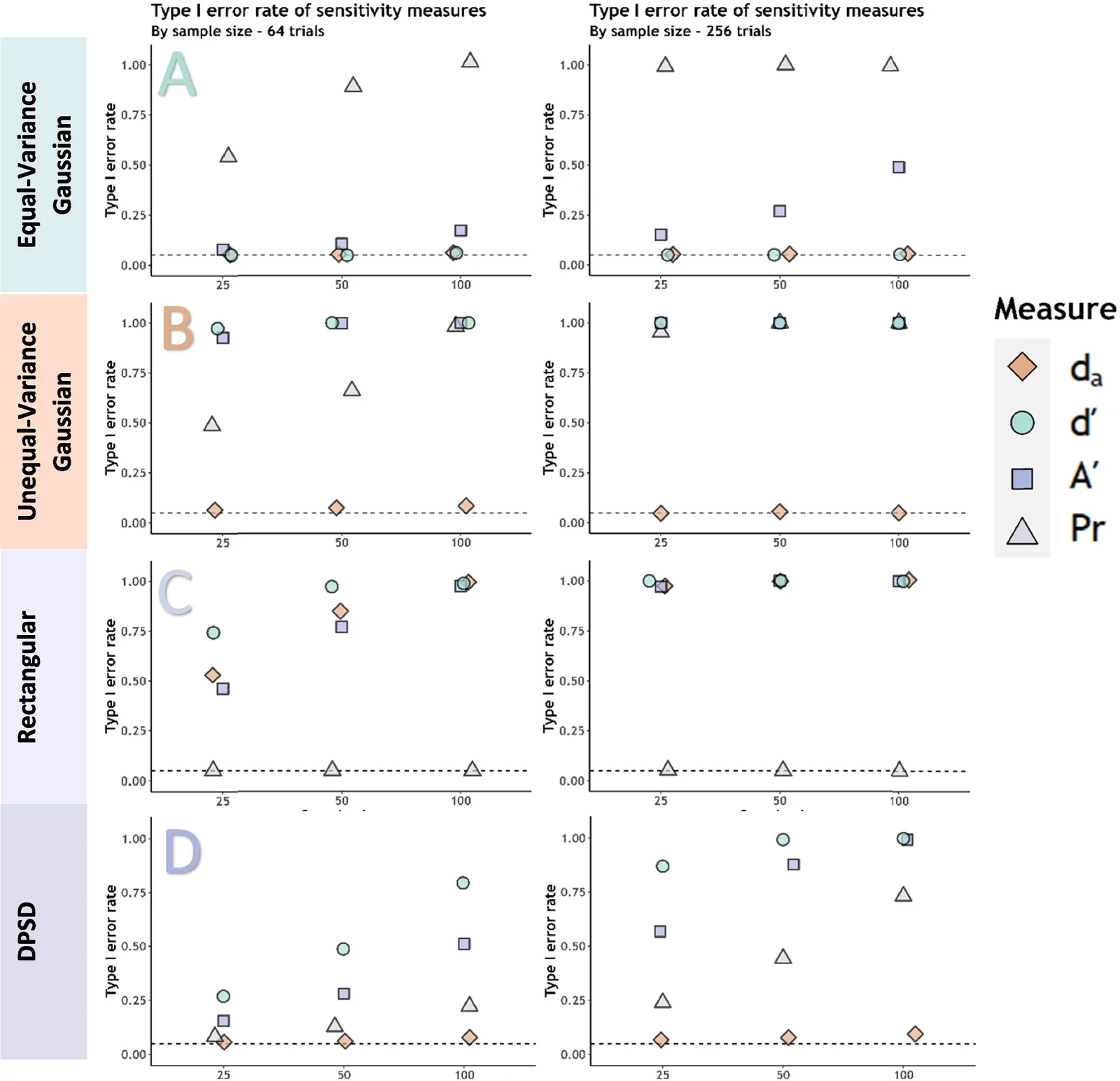
Type I error rates of *P*_r_, *A*′, *d*′, and *d*_a_ as a function of sample size for small (left column) and large (right column) numbers of trials (64 and 256 trials per condition, respectively). The assumed distributions were Gaussian (equal variance, panel **A**; unequal variance with SD ratio = 0.8, panel **B**), rectangular (panel **C**) or DPSD (panel **D**) distributions. For Gaussian distributions, the spread of criteria was neutral and wide. The distance between distributions was 1 SD (relative to the lure distribution), and the size of shift in criteria between conditions was 0.5 SDs (relative to the lure distribution). For rectangular distributions, the spread of criteria was neutral and narrow. The distance between distributions was 0.37 SDs (relative to the lure distribution), and the size of shift in criteria between conditions was 0.08 SDs (relative to the lure distribution). For DPSD, the spread of criteria was also neutral and wide, and the size of shift in criteria between conditions was 0.5 SDs (relative to the lure distribution). The proportion of recollection was 0.17, and the distance between the familiarity target distribution and lure distribution was simulated as 1 SD (relative to the lure distribution). The dashed line indicates α =.05

Like increasing the sample size, our simulations show that increases in the number of trials were associated with increased Type I error rates. Figure 9 presents T1ERs of the different measures as a function of sample size (25, 50, or 100 participants per experiment) for both fewer trials (64 per condition) and more trials (256 per condition), as compared to the simulations depicted in Fig. 7. Like the patterns found with increases in sample size, when Gaussian distributions were assumed, T1ERs of *d*_a_ were curtailed at 5%. In contrast, T1ERs increased up to 100% for SPSMs (*P*_r_, *A*′, and *d*′) as the number of trials increased.

For SPSMs, we again replicated Rotello et al. (2008). As observed in Fig. 9, under all scenarios for which T1ERs were higher than 5%, an increase in the error was associated with an increase in both the number of trials and the sample size. For example, when computing TIERs of *d*′ for unequal-variance simulations (Fig. 9, panel B), T1ERs unfailingly reached 100% for simulations with 256 trials and sample size of 100 participants.^18^

The one measure that did not demonstrate an association between number of trials and T1ERs for Gaussian distributions was *d*_a_ (see Fig. 9, panels A and B). The only scenarios for which higher T1ERs were observed for *d*_a_ occurred when a higher number of trials were simulated under rectangular distributions. For the DPSD simulations, *d*_a_ only showed a positive association between the number of trials and T1ERs under a specific set of parameters (e.g., large shift in criteria between conditions or a large proportion of recollection trials). Again, this pattern of results is predicted in that the measurement model of *d*_a_ is incongruent with both a data-generating model of the 2HT model (i.e., rectangular lure and target underlying distributions) and the data-generating model predicted by DPSD. Still, the level of discrepancy between the measurement model of *d*_a_ is smaller for the DPSD data-generating model than the 2HT data-generating model. Therefore, high T1ERs emerged for the DPSD model only when the smaller differences achieved statistical significance, that is, in high-powered conditions (i.e., a greater number of trials or participants).

However, more modest increases in T1ERs for *d*_a_ were observed under unequal-variance Gaussian distributions when two *conjoint* conditions were met: a low number of trials *and* a large sample size. Notably, T1ERs of *d*_a_ reached approximately 10% when the sample size was 100 (the largest simulated sample size) with 64 trials per condition (the fewest simulated number of trials; see left column of Fig. 9, panel B). Still, the sample size of 100 did not produce a high T1ER when the number of trials per condition was higher (128 or 256; see Fig. 7, top-right panel and Fig. 9, right column, panel B, respectively). In the Discussion, we provide an explanation for this pattern of results. For now, consider the idea that a low number of trials can result in a *z*ROC slope (*S*) that deviates from the true SD ratio, steering the measurement model away from the data-generating model. In turn, a large sample can increase the power to detect differences between this measurement model and the data-generating model. We will return to this issue in the Discussion.

It is important to note that even when T1ERs of *d*_a_ grew higher than the acceptable 5%, the effect of number of trials was opposite in direction to the one observed with SPSMs. When the simulated distributions were Gaussian, for *d*_a_, a higher number of trials led to a lower T1ER (see Fig. 9, panels A and B). In contrast, the SPSMs showed higher T1ERs for a higher number of trials (see Fig. 9, panel B).

### Type I error rates of *AUC*_g_

**Fig. 10:**
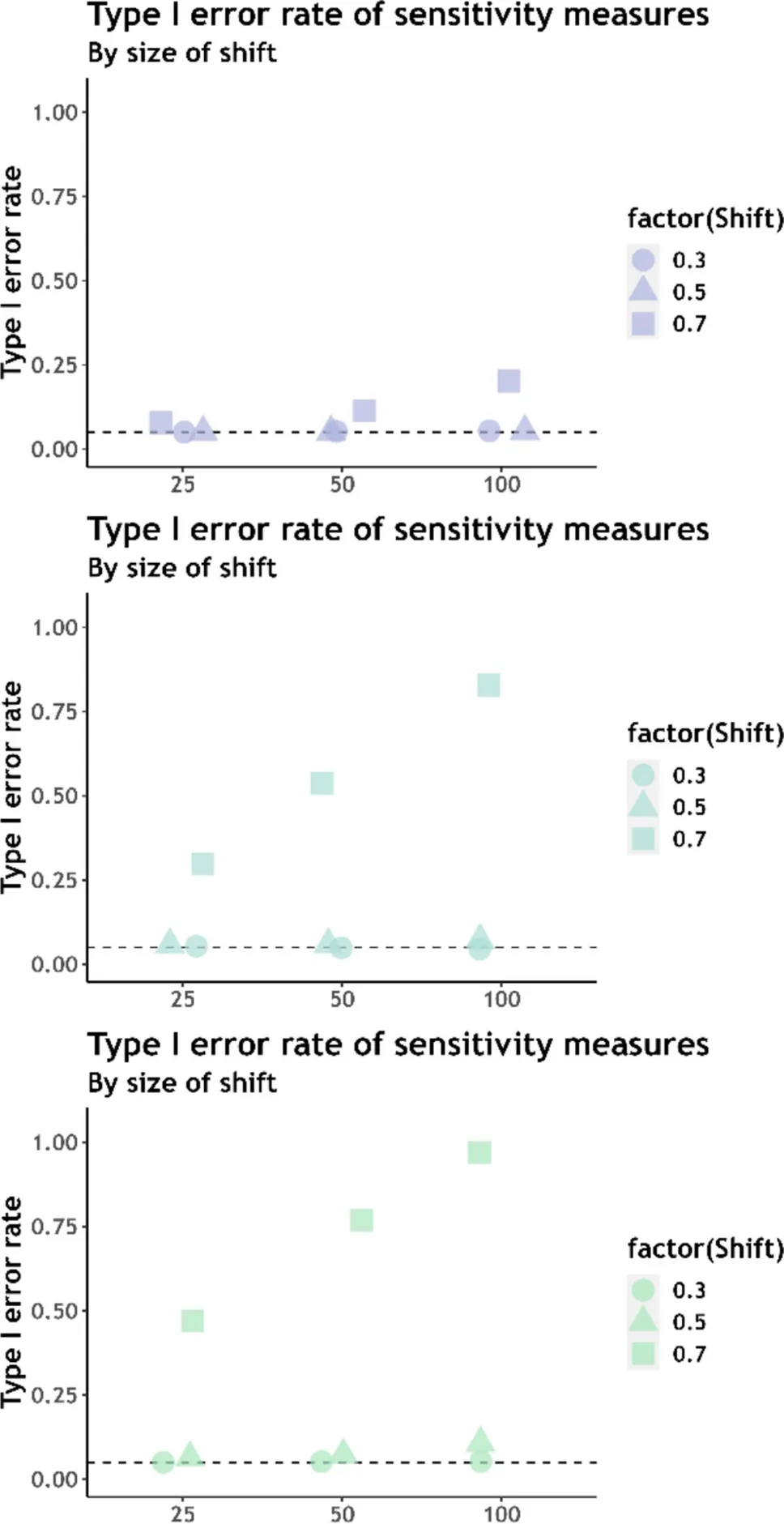
Type I error rates of *AUC*_g_ as a function of sample size for different sizes of shift in criteria between conditions (circles, triangles and squares represent 0.3, 0.5, and 0.7 SDs relative to the lure distribution, respectively). Simulated number of trials was either 64 (top panel), 128 (middle panel), or 256 (bottom panel) trials per condition. The assumed distributions were unequal-variance Gaussian with SD ratio = 0.8, the spread of criteria was neutral and wide, and the distance between distributions was 1 SD (relative to the lure distribution). The dashed line indicates α =.05. See Fig. 3 for an explanation of why *AUC*_g_ is bias-dependent

Figure 10 shows T1ERs of the *AUC*_g_ as a function of sample size and the size of shift between conditions for different numbers of simulated trials. Although this measure makes no parametric assumptions, our results revealed that *AUC*_g_ was not bias-independent. To understand this finding, please refer to Fig. 3.

When the difference in bias between conditions (i.e., the size of shift in criteria) was large enough (i.e., 0.7 SDs relative to the lure distribution), T1ERs were consistently higher than 5% when using *AUC*_g_ as a measure and were positively correlated with the sample size and number of trials (see Fig. 10). For the large shift in criteria, when we simulated 100 participants, each with 256 trials per condition, T1ERs of *AUC*_g_ reached 100% (or close to 100%) for any type of criteria placement and any data-generating model (see Appendix A).

Even when the difference in bias between conditions was smaller (i.e., 0.5 SDs relative to the lure distribution), T1ERs of *AUC*_g_ were higher than 5% for many experimental scenarios. This pattern of results shows that *AUC*_g_ is bias-dependent and, therefore, an invalid measure of sensitivity in recognition memory.

### The effect of extent of participants’ exclusion

As shown in our results, our simulations showed promise regarding the validity of *d*_a_ (see Appendix A)^19^. However, one additional pattern is worth mentioning. When the data-generated model assumed Gaussian distributions, T1ERs were higher than 5% when a large number of simulated participants were excluded from the *t*-test analyses. As described in the Method section, simulated participants’ data were excluded from analysis when fewer than three operating points could be extracted in either of the two conditions. The observation of higher T1ERs when more data were excluded was most often identified in simulations with fewer trials. As a result, the *retained* sample sizes (i.e., the functional sample size after exclusions) were smaller than the *simulated* sample sizes (i.e., the sample size that was set in the computer simulation; the nominal sample size). See Appendix E for a detailed tabulation of the retained sample sizes as a function of the simulated samples sizes and as a function of number of trials across participants.

**Fig. 11:**
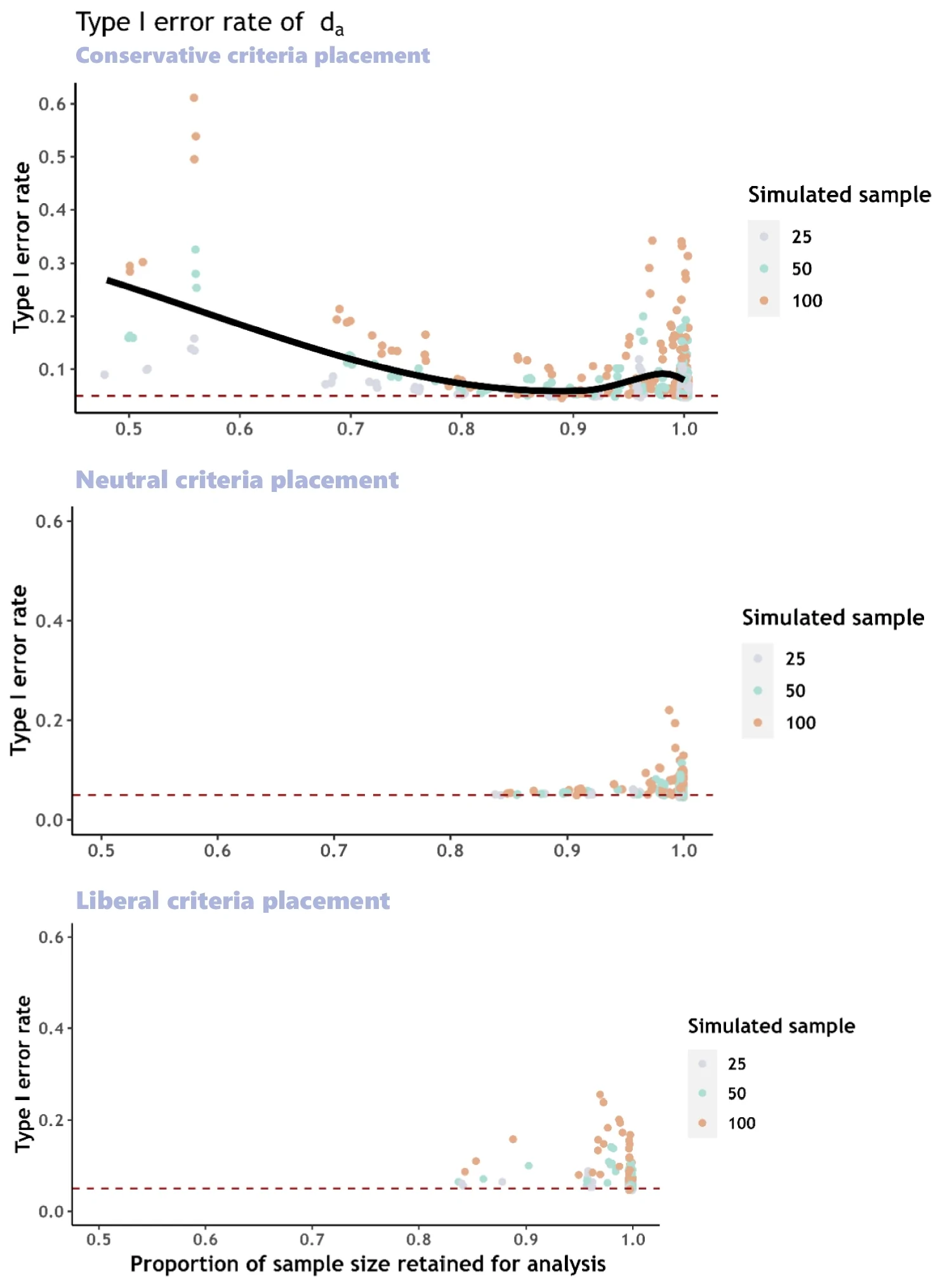
Type I error rate of *d*_a_ as a function of the proportion of sample size retained for analysis, out of the simulated sample size. All the simulations described in this figure are Gaussian. Conservative criteria placements are presented in the top panel, neutral criteria placements are presented in the middle panel, and liberal criteria placements are presented in the bottom panel. Different sample sizes are depicted as different colors. The dashed line indicates α =.05

Figure 11 depicts the T1ER as a function of the proportion of the retained sample size (out of the simulated sample size), the simulated sample size, and the placement of the criteria. A decrease in the proportion of the simulated sample size that was retained for analysis was associated with a higher frequency of T1ERs that exceeded 5%, mostly under the conservative criteria placement (Fig. 11, top panel). High T1ERs were typically observed for conservative criteria because this placement allowed a relatively smaller proportion of simulated samples to be retained for analysis. It is important to note that even when considering simulations where the mean retained sample size was small, higher T1ERs were observed only for a small number of those simulations; most simulations were associated with T1ERs of approximately 5%. In fact, most simulations did not have exclusions in any of the 10,000 iterations^20^.

In Appendix E, we investigated the retained sample size, presenting the effects of simulated sample size and the number of simulated trials per condition on the median size of the retained sample size. In Appendix F, we explored an additional pattern of results, testing the computation of* S* for different Gaussian simulations. We showed that the overall similarity between *S* and the true SD ratio is high, and how decreases in retained sample size were associated with estimates of *S* that deviated to greater extents from the true SD ratio. We return to these results in the Discussion.

## Discussion

We ran 1,962 Monte Carlo simulations to explore the validity of different sensitivity measures under the assumptions of four different data-generating models. The validity of a measure was defined as bias independence, namely, that significant differences in sensitivity between two iso-sensitive conditions did not exceed the acceptable alpha level when the conditions differed only in bias. The difference between the two conditions was tested using a within-participant *t*-test. If a measure is bias-independent, it should yield a T1ER of approximately 5%—our dependent variable. We investigated three SPSMs: *P*_r_, *A*′, and *d*′, as well as two multi-point measures: *AUC*_g_ and *d*_a_.

All five sensitivity measures were investigated assuming one of four data-generating models: equal- and unequal-variance lure and target Gaussian distributions, rectangular lure and target distributions, and a mixture model (DPSD) that assumes recognition is mediated by a discrete recollection process on some trials and a continuous familiarity process on the others. These simulations demonstrated that, of the measures considered, *d*_a_ is the best contender for indexing sensitivity in recognition memory: it was associated with approximately 5% Type I error rates observed under most conditions, and displayed the lowest error rates under the remaining conditions.

Our strategy for computing *d*_a_ required an estimate of the lure-to-target SD ratio using *S* (the slope of the *z*ROC curve). This algorithm is based on the estimation of *S* for each participant (using the mean value across the two conditions). Our algorithm for computing *S* entailed including in the computation only participants with at least three operating points from a total of five possible points. We also considered several alternative algorithms for estimating the SD ratio, all of which yielded higher T1ERs (Appendix C).

### *d*_*a*_

*For Gaussian lure and target distributions, T1ERs of d*_*a*_* were approximately 5% across different sample sizes, number of trials, response criteria, and distances between distributions.* Importantly, the 5% T1ERs were found when the data-generating model was unequal-variance Gaussian distributions (the UVSD model, the data-generating model that many researchers argue best describes recognition memory). If, as believed by many researchers, recognition memory is indeed mediated by lure and target Gaussian distributions with an average SD ratio of 0.8, then *d*_a_ may be the much-anticipated solution to the measurement crisis in memory (see Figs. 7 and 9).

As expected, *d*_a_ was not valid (i.e., did not exhibit bias independence) for rectangular distributions. Finally, for the DPSD-based simulations, *d*_a_ yielded T1ERs approximating 5% when using parameters from which the data structure (the implied ROC curves) closely resembled that of the UVSD model. For parameters whose data structure deviated from that of UVSD, higher T1ERs were found. At bottom, our simulations revealed *d*_a_ to be a bias-independent measure under most scenarios of the signal detection models (equal-variance signal detection and UVSD models) and under many scenarios of the DPSD model.

An important caveat is in order here. We only investigated the generic and commonly used versions of the continuous Gaussian models (the equal-variance signal detection and UVSD models, both assuming Gaussian distributions) and one generic and commonly used version of a mixture model (the DPSD model). However, there are other possible assumptions within the families of continuous evidence and mixture models. For example, rather than assume Gaussian distributions, as the UVSD model does, one could assume another continuous distribution. Several distributions have been investigated, including the Gumbel, logistic, Weibull, lognormal, exponential, and gamma distributions (see Berry & Shanks, 2024). Alternate mixture models have also been proposed, including the continuous dual-process model (Wixted & Mickes, 2010b), the mixture signal detection (MSD) model (DeCarlo, 2002), the Moran and Goshen-Gottstein (2015) model, and the VRDP model (Onyper, Zhang, & Howard, 2010). Although our simulations fared well for the most prototypical versions of the two families of models, there is no guarantee that the specific derivatives, each of which makes different assumptions, will likewise fare well. Given the multitude of alternative models, we can only hope that proponents of those specific models will explore whether *d*_a_ remains bias-independent under their distributional assumptions.

### SPSMs

Our simulations demonstrated the failures of common SPSMs (*P*_r_, *A*′, and *d*′) for almost all data-generating models. These failures of SPSMs in most simulations replicated the failures reported in previous, more limited simulations (Rotello et al. 2008; Schooler & Shiffrin, 2005). Our results also replicated the finding that for all SPSMs, T1ERs increased with more trials or with larger samples, reaching levels of 100% T1ERs. New in the simulations was the use of the DPSD model as a data-generating model. 

The only exceptions to the failures of SPSMs occurred in simulations that used data-generating models congruent with the measurement model of a specific measure. For example, when the data were generated using the equal-variance Gaussian model, the T1ERs for *d*′ were low because *d*′ is congruent with that model. Similarly, when the data were generated from the 2HT model using rectangular distributions, premised by *P*_r_, the T1ERs for *P*_r_ (but not the other SPSMs) approximated 5%.

Using a sensitivity measure that is incompatible with the data-generating model can be thought of as analogous to using the wrong measure of central tendency when performing a *t*-test. For example, although means and medians are highly correlated, replacing means with medians will yield invalid conclusions in a *t*-test.

We found that all the SPSMs we tested lack robustness to violations of their measurement model assumptions. Stated differently, there is no preferred choice among SPSMs (i.e., none that are “less bad”), because the rank ordering of their T1ERs changes across the specific and detailed parameter settings in the simulations. We did observe some differences in T1ERs between SPSMs, depending on the parameter values we manipulated (e.g., criteria placement and distance between distributions. See Appendix A). However, apart from the exceptions specified above, no single SPSM was consistently associated with lower T1ERs. If the true data-generating model of recognition memory is either the UVSD model or the DPSD model, then all the SPSMs we tested can lead to invalid conclusions at more or less the same rates.

Like Rotello et al. (2008), we too found a positive association for SPSMs between the T1ERs and sample sizes. This association is alarming. To increase statistical power, researchers recruit large samples, as recommended following the replication crisis (Open Science Collaboration, 2015). Our results suggest that large sample sizes, while increasing power, can also increase the rate of Type I errors in recognition memory (or other similar tasks) if invalid measures are used. *These results confirm that collecting more data (e.g., by having a larger sample size or more trials) will not improve the validity of these SPSMs.* Therefore, identifying a measure of sensitivity that has a measurement model congruent with the true data-generating model of recognition memory is vital for the ability to sustain high-powered, reproducible, and (most importantly) valid recognition memory research.

### *AUC*_g_

An important novel finding in our new simulations was that the non-parametric measure, *AUC*_g_, is not independent of bias. We found a positive relationship between the magnitude of the bias shift and T1ERs. Additionally, and similar to the results for the SPSMs, the T1ERs for *AUC*_g_ rise when experiments include more trials and/or are conducted with larger sample sizes.

As a non-parametric measure, *AUC*_g_ is often considered a perfect solution to the measurement crisis. If the crisis is grounded in the incongruity between the data-generating model and the measurement model, a measure that does not have a measurement model would ostensibly be bias-independent. Alas, the manner in which *AUC*_g_ is computed results in an invalid measure that confounds sensitivity with bias (as demonstrated in Fig. 3). To reiterate, *when the difference in bias was large and more data was collected (i.e., more trials or larger sample size), AUC*_*g*_* reached a T1ER of 100% for all data-generating models*.

### The use of confidence ratings

To compute *d*_a_, confidence ratings were needed to estimate the SD ratios with *S*. The calculation of *S* is not possible with binary old–new judgments. Alas, researchers might be reluctant to move from binary responses to confidence responses. Indeed, one article has presented arguments that maintaining the placements of multiple criteria, as required by confidence rating, yields noisy criteria in comparison to the single criterion required in binary judgments (Benjamin et al., 2009). Still, a re-analysis of these arguments found the effect of noisy placement to be of very low magnitude, bearing no price for the use of confidence ratings (Kellen et al., 2012). We argue that the need for confidence ratings should not discourage researchers from using *d*_a_, the only measure that shows promise of being a bias-independent measure of sensitivity. Instead, we believe our simulations show the undeniable value of making the modest effort to collect confidence data: valid inferences can be made about experimental data.

Once confidence ratings are available, researchers who are equipped with the relevant expertise might be tempted to suggest modeling techniques as a means to estimate sensitivity. We reiterate that the route of model fitting is not free of assumptions. Fortunately, all researchers can easily use *d*_a_ to analyze their data. We endorse *d*_a_ because it is a simple, straightforward measure that can be used by all researchers, and our simulations point at its high validity.

### Estimation of the lure-to-target SD ratio

Most Gaussian simulations revealed 5% T1ERs with *d*_a_. We now turn to discuss the higher false discovery rates that were found under two specific scenarios. Common to both scenarios is that circumstances reduced the consistency of *S* as an estimator of the SD ratio. That is, in those scenarios, *S* was a biased estimator that did not approach the true SD ratio when more data was available.

In the first scenario, T1ERs for *d*_a_ were greater than 5% for large sample sizes (*N* = 100) when fewer trials (64 trials) were simulated (see Appendix A). The high T1ERs under these circumstances can be explained in the following way. As we have repeatedly noted, for a measure to be valid and have 5% T1ER, the measurement model must be congruent with the data-generating model. One element of the measurement model of *d*_a_ is the SD ratio, estimated by *S*. If participants provide few responses, for example, because there are relatively few trials per condition, then the estimate of *S* is less likely to approximate the true SD ratio (see Fig. 7 and Appendix A; also, Macmillan et al., 2004). Consequently, the measurement model will likely deviate from the data-generating model. Though the resulting discrepancies may be small, they can be consequential in highly powered experiments. This is exactly the situation at hand: experiments with larger *N* (= 100) and fewer trials (= 64) are more likely to result in Type I errors. It is for this reason, that T1ERs higher than 5% were found for the conjunction of large sample size with few trials (see Appendix A).

The remedy for the large T1ERs found in large sample sizes might be as simple as having more trials. If a small number of trials lead highly powered experiments to show false discoveries at a rate higher than 5%, a larger number of trials should reduce the T1ER back to 5% because *S* will provide a more accurate estimate of the true SD ratio. This in turn should result in greater congruency between the data-generating model and the measurement model. Therefore, T1ERs will likely be reduced to the desired 5%. Indeed, our simulations generally support this conclusion and the recommendation to simply increase trials: We found that when the number of trials was higher (128 or 256 trials), T1ERs were approximately 5% for *d*_a_, even with large sample sizes (see Results, Figs. 7 and 9).

Our conclusion is that *d*_a_ can be used in large-sample experiments so long as the number of trials each participant undergoes is large, closer to 128 than to 64. Fortunately, this is already the case for most recognition memory studies where researchers typically run tasks with considerably more than 64 trials per condition. If circumstances require a limited number of trials, perhaps because the relevant study items are a small set (e.g., taboo words), then we urge careful consideration of other relevant factors such as overall criterion placement and spread that may also influence Type I errors. The detailed simulation data provided through the OSF repository (Appendix A) offer a resource to guide researchers in these less common situations.

The second scenario under which high T1ERs for *d*_a_ were observed was when the proportion of the original sample size that was retained for analysis (i.e., the proportion of participants with at least three operating points in both conditions) was considerably smaller than the simulated sample size (see Fig. 11). This was found mostly for conservative criterion locations. The higher T1ERs seem, again, to be the outcome of the less accurate parameter estimation (*S*). As before, this pattern was observed primarily for simulations with a small number of trials.

These two exceptions notwithstanding, we argue that *d*_a_ is a better alternative than SPSMs for measuring sensitivity and is, in fact, an excellent alternative. This measure is bias-independent for experiments with many trials, an attribute of the experiment that is often under the researcher’s control. When such experiments are run, a cautionary signal for T1ERs higher than 5% is if a large portion of participants have fewer than three operating points. Results with many such exclusions should be viewed with caution and may suggest that a redesign of the experimental procedure is in order.

Importantly, the use of *S* introduces a data-based estimation of an unknown parameter value (the true SD ratio) into the calculation of *d*_a_. To be clear, there is a cost associated with using an estimate rather than the true (unknown) population parameter. Our data reveal that knowing the true value of the population parameter and using it in the calculation of the measure yields reduced levels of T1ERs. This was the very finding observed in our equal-variance Gaussian distributions simulations, wherein the population parameter of the SD ratio equaled 1. We compared T1ERs for *d*′—a measure with a built-in assumption of SD ratio equal to 1—to *d*_a_ that was computed based on a data-based estimation of the SD ratio. The comparison revealed that for most of the equal-variance Gaussian simulations, *d*′ was associated with slightly lower T1ERs than *d*_a_. The reason is simple: Any estimation inevitably yields slightly inaccurate values for some participants in each simulated experiment. Unfortunately, researchers do not have access to the true SD ratio. Most importantly, our simulations demonstrate that whenever the SD ratio is unknown—the case for all the empirical experiments using recognition memory tasks and perhaps any experiment using any single-intervals task—*d*_a_ is the most valid choice for indexing sensitivity.

In our simulations, the values of the SD ratio and their estimates (*S*) were approximately the same. In most experimental scenarios, the mean *S* values deviated from the true SD ratio by about 0.05 or less (see Appendix F). The largest deviations of mean *S* from the true SD ratio were observed in simulations in which the placement of criteria was conservative. Indeed, conservative criteria placement simulations were also the simulations in which most cases of T1ER higher than 5% were observed for *d*_a_ (see Results, Fig. 11).

Presumably, because of the location of criteria, conservative placements yielded more FAR = 0% than other placements, yielding fewer than five operating points for many participants and therefore a less accurate estimations of the SD ratio—that is, values of *S* that were further from the true SD ratio^21^. As we have seen, less accurate values of *S* lead to a larger discrepancy between the simulated data-generating model and the measurement model of *d*_a_ (which requires *S*), resulting in higher T1ERs.

Nevertheless, for the vast majority of our simulations, the values of *S* were similar to the true SD ratio and thus provided a good estimate for the computation of *d*_a_. This result is not surprising in that the simulations did not include any noise and as such were ideal for the estimation of any parameter, including the true SD ratio. Future research is required to test the estimation of *S* using empirical data, where the control over noise is limited.

### The limitations of simulations

To evaluate the implications of our simulations to future recognition experiments, we wish to point out an asymmetry in the potential of simulations to advance the validity of any measure. Simulations provide a robust tool for showing a measure to be *invalid*, in our case, establishing that all of the tested SPSMs as well as *AUC*_g_ are not bias-independent. However, to establish that a measure—any measure—is *valid* and bias-independent, simulations are not enough.

For the SPSMs and *AUC*_g_, our simulations represent possible experimental scenarios under which T1ERs are higher than 5%. Currently, for recognition memory research, there is convincing evidence that such experimental scenarios do exist, most notably, there is a strong possibility that the lure and target distributions are indeed at least approximately unequal-variance Gaussian. Because of the severe consequences of these high Type I error rates, which impair our ability to draw appropriate inferences from our data and thus slow the advancement of our science, simulation-based evidence should be sufficient for researchers to renounce those measures and seek alternatives.

For *d*_a_, however, 5% T1ERs in Gaussian distribution simulations are not sufficient to prove that this measure is in fact valid. Simulations always require assumptions to be made. Specifically, in our simulations, we knowingly made assumptions regarding the data-generating model of recognition memory. As such, we ran simulations based on Gaussian (both equal and unequal variance) and rectangular distributions, as well as simulations assuming the DPSD model. *d*_a_ only showed consistently low T1ERs when data were simulated from Gaussian distributions. The debate in the recognition literature regarding the data-generating model of recognition memory (e.g., Wixted, 2007; Parks & Yonelinas, 2007; Mickes et al., 2007; Rouder et al., 2010; Wixted & Mickes, 2010a) is ongoing, and results from our simulations are orthogonal to the question of the true recognition memory data-generating model. Thus, for example, our simulations reveal that if the true data-generating model involves rectangular lure and target distributions, *d*_a_ will not be valid. Because we have only indirect access to the true data-generating model in recognition memory, the only way to guarantee the validity of *d*_a_ will be by analyzing real-world empirical data.

Many aspects of the data generation process in our simulations were based on assumptions that we know are overly simplistic. For example, we assumed that all participants in our simulated experiments had exactly the same underlying memory distributions, in clear violation of empirical evidence. Similarly, the confidence ratings in each trial were sampled independently. However, confidence carry-over effects have been observed empirically, such that participants’ confidence on trial *N* is correlated with their confidence on trial *N* – 1 (Kantner et al., 2019). In terms of our simulations, accounting for confidence carry-over effects would entail adding some amount of noise to the criterion locations, which we did not do. Thus, we do not know the extent to which such carry-over effects will affect the validity of *d*_a_.

Like the carry-over effect, an empirical setting may involve many other effects we are unaware of that nevertheless may impact the validity of *d*_a_. Because we cannot simulate what we are unaware of, we cannot know the extent to which these effects impact the validity of our measures. The only way to prove the validity of *d*_a_, beyond any known and unknown effect in recognition data, is to put it to the test using empirical data from iso-sensitive conditions.

### Power

One possible question about our embrace of *d*_a_ as a sensitivity measure is whether its low rate of Type I errors is due to an inability to detect any change at all. That is, *d*_a_ may simply be associated with high levels of Type II errors (low power). Though the frequency of *d*_a_ in the literature is still extremely low (see Fig. 1), several articles did report using this measure. Importantly, when using *d*_a,_ significant differences in sensitivity have, in fact, been found (e.g., Verde & Rotello, 2004b; Masson & Rotello, 2009; Macmillan et al., 2021; Yüvrük & Kapucu, 2022; Trzewik et al., 2024). Although these articles offer preliminary evidence that *d*_a_ can detect genuine differences, they do not definitively establish its statistical power. Like our current investigations of T1ERs, future research must apply systematic investigations to the rates of Type II errors.

### Previous recognition research: Guidelines for interpretation

In the current investigation, we propose a possible valid measure of sensitivity for all future recognition memory research (i.e., *d*_a_). In this article, we made the bold proposition that “*Many recognition studies that used standard measures and reported differences in sensitivity may have reached conclusions that are not supported by their data.”* This claim calls into question thousands of peer-reviewed journal articles, no doubt causing many readers to wonder whether it is ever possible to reach valid conclusions regarding sensitivity from previous results based only on binary responses and indices such as *d*′ or *P*_r_.

A reevaluation of the prior literature requires a careful inspection of each individual experiment, considering potential bias differences across conditions. We now describe two scenarios that, when observed, would lessen our concern that the effects are mediated by bias rather than sensitivity, even when invalid measures were used. These scenarios are exceptions to our general concern that effects reported with invalid measures may reflect bias rather than sensitivity. We also consider a third such scenario in which the question of whether bias shifts can explain the data is still a matter of debate.

First, studies that demonstrate a *mirror effect* (Glanzer & Adams, 1985) are likely to be reporting a true effect of sensitivity. The mirror effect refers to a reverse association—an interaction—between participants’ HRs and their FARs. Specifically, a mirror effect is found when a condition that is associated with higher HR is the also the conditions with the lower FAR. In such cases, an explanation based on bias is implausible, because a higher HR, interpreted as a liberal criterion, cannot simultaneously induce a lower FAR, which is interpreted as a more conservative criterion.

Still, a pattern of HRs and FARs that fits a mirror effect does not guarantee that *only* sensitivity changes across conditions. If bias also changes, the magnitude of the difference in HR and FAR across conditions would be affected. In such cases, even though a defensible argument would posit that that the conditions differ in sensitivity, the effect size of the sensitivity difference cannot be computed based on the HR–FAR interaction. Thus, any interpretation of the finding would remain more limited than findings derived from a bias-independent measure of sensitivity (i.e., *d*_a_).

Second, a specific pattern of results can be evaluated using an understanding of the ROC and *z*ROC curves. Because each of the SPSMs assumes a specific *z*ROC curve, the discrepancy between the *z*ROC curve assumed by the measurement model and the *z*ROC curve assumed by the data-generating model can help in evaluating if an effect of bias created a significant difference between conditions.

**Fig. 12:**
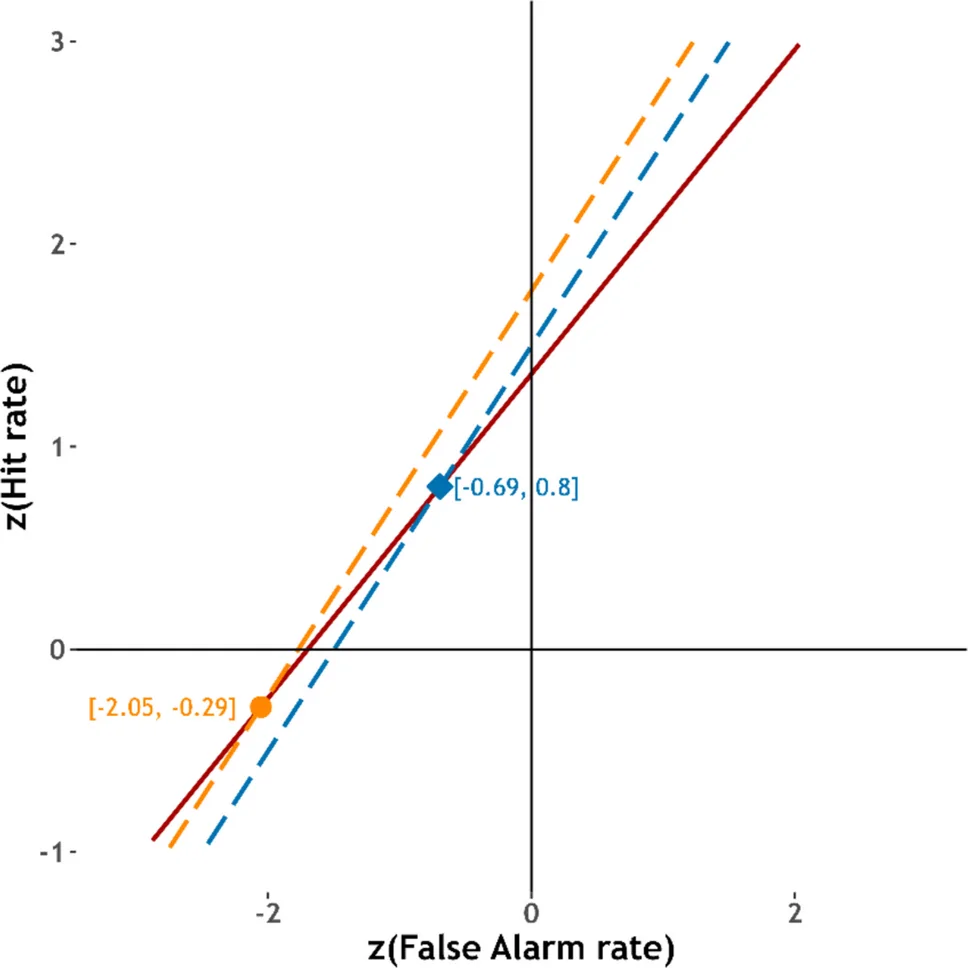
Example of a bias effect in *d*′. The orange circle marks the [*z*FAR, *z*HR] values measured in Condition A, and the blue diamond marks the [*z*FAR, *z*HR] values measured in Condition B. The red line represents the *z*ROC curve fitting a UVSD data-generating model (SD ratio of 0.8), with a sensitivity level of 1.5 SDs (relative to the lure distribution). The orange and blue dashed lines represent the *z*ROC curve that fit an equal-variance signal detection measurement model (assumed by indexing sensitivity with *d*′) for Condition A and B, respectively. Because the slope of the blue and orange curves is larger than the slope of the red curve (i.e., *d*′ assumes a *z*ROC slope of 1, but the actual slope is 0.8), the more conservative, orange operating point (i.e., Condition A) produces a higher value of *d*′, as represented by the higher intercept of the ordinate

For example, consider the comparison of two conditions in a recognition experiment. Condition A yields a mean HR of 0.388 and a mean FAR of 0.02. Condition B yields a mean HR of 0.789 and a mean FAR of 0.244. In this example, the conditions are iso-sensitive, and the true data-generating model is the UVSD model (with an SD ratio of 0.8). As commonly observed in the previous literature, if we use *d*′ to measure sensitivity, it will produce the following values: Condition A, 1.769; Condition B, 1.497. Importantly, because there is no effect of sensitivity in this example, the more conservative condition (i.e., Condition A) necessarily produces the higher value of *d*′ (see Fig. 12).

The conservative *d*′ produces higher values because the true, UVSD-based, *z*ROC slope is smaller than the slope assumed by *d*′. Thus, as Fig. 12 depicts, a higher-slope line that must include a more conservative operating point (i.e., the orange circle, see Fig. 12) will necessarily intercept the ordinate on a higher point, corresponding to a higher value of sensitivity. Importantly, if the condition with higher sensitivity index (i.e., *d*′) is also *more liberal*, the possibility of a bias interpretation for the results is less likely.

Therefore, because we know that a more conservative bias increases the value of *d*′ when the slope of the *z*ROC is less than 1, it is possible to evaluate whether bias can explain the significant difference in *d*′ by considering which condition has a more conservative bias. Importantly, this is a rule of thumb that can only help us make an intelligent guess regarding previously studied research. By using this heuristic, researchers can identify previous studies that are more likely to have discovered a true effect of sensitivity. Here again, we cannot estimate the size of this effect.

Third, specific designs of the recognition memory task are arguably not threatened by a bias confound. These designs comprise experimental conditions that differ during encoding by learning methods (e.g., a repetition or levels-of-processing manipulation). At test, a common pool of lures defines the FAR across conditions. Because the FAR is based on the same set of lures for all the experimental conditions, it is often referred to as a *common FAR design*. Some evidence suggests that participants are neither willing nor able to shift decision criteria across encoding conditions within a single list, even when condition-specific cues are provided for lures, such as color cues (Stretch & Wixted, 1998; Verde & Rotello, 2007; Hicks & Starns, 2014). This design often results in HRs that differ across conditions while FARs remain approximately equal (i.e., the absence of a mirror effect), thus it has sometimes been called *the mixed-list-strength-no-mirror effect,* an accurate, if awkward term (Hilford et al., 2019). If a within-list criterion shift is not possible, then bias shifts cannot explain the difference between experimental conditions.

Importantly, however, there is a debate in the literature regarding within-list criterion shifts in these mixed-list designs. For example, both Hilford et al. (2019) and Franks and Hicks (2016) showed that sufficiently salient discriminanda (e.g., sensory modality or response keys) produce measurable FAR differences even under mixed-list designs. These findings suggest a within-list criterion shift (i.e., bias shift) interpretation: Participants might shift their response bias implicitly across encoding conditions that are intermixed at test based on differences in processing fluency, for example (Whittlesea et al., 1990; Whittlesea & Williams, 2000). It remains unclear whether and to what extent response bias can provide an alternative explanation for experiments based on these designs (for a more comprehensive discussion of these issues, see Danino & Goshen-Gottstein, 2026).

Unfortunately, it is difficult to assess what fraction of the data patterns in the recognition literature fall under the three exceptions described above. The inconvenient truth is, therefore, that there is an unknown percentage of studies from which we cannot draw any conclusions regarding sensitivity based on their data. Those studies may have reported a true effect of sensitivity, but we are unable to confirm or deny the existence of such an effect without applying a valid, bias-independent measure to the data.

Thus, the only research that can emerge completely unscathed is future research. Memory researchers aiming to make any advances to the field using recognition tasks must change their practices and replace invalid measures with measures such as *d*_a_. This entails that reviewers recommend rejecting manuscripts that continue to use SPSMs and that editors embrace such decisions.

While future research is needed to establish the validity and power of *d*_a_, our current investigation points at a promising direction in support of this lesser-known sensitivity measure for indexing accuracy in recognition tasks. Importantly, as a replication of previous simulations (Schooler & Shiffrin, 2005; Rotello et al., 2008), our results also confirm the importance of choosing a valid measure, not only in recognition memory but for other cognitive tasks as well. The choice of a measure must always consider the data-generating model of the task, and the fit between the data-generating model and the measurement model, so conclusions can be drawn from valid results. The choice of a valid measure is an essential and understudied aspect of research design and analysis.

## Supplementary Information

Below is the link to the electronic supplementary material.

Supplementary file1 (DOCX 613 KB)

Supplementary file2 (DOCX 56.3 KB)

Supplementary file3 (DOCX 19.0 KB)

Supplementary file4 (DOCX 29.6 KB)

## Data Availability

Both the data generated during the current study and the simulations script we used are available through the OSF repository: https://osf.io/d5jub.

## Footnotes

1 The diagnosticity ratio is defined as the hit rate divided by the false alarm rate (for a meta-analysis, see Steblay et al., 2011). The terms hit rate and false alarm rate are defined below.

2 Our use of independent does not imply stochastic independence (for a comprehensive analysis of functional and stochastic independence, see Moran & Goshen-Gottstein, 2015).

3 We describe a review of the measures used in 7 years of published articles in Springer Nature journals (see Fig. 1). We were surprised to learn that 7.3% of articles did, in fact, choose HR to index sensitivity.

4 The only known measure that is non-parametric, and therefore does not entail assumptions regarding the population, is the geometric area under the ROC curve (AUC_g_). In our simulations, we also tested AUC_g_ (see “Multi-point sensitivity measures and ROCs”)*.*

5 In the following sections, we specify scenarios under which specific measures do turn out to have low T1ERs. We then explain why we believe these scenarios likely do not reflect the processes underlying recognition data.

6 Notably, several articles used mathematical modeling rather than any sensitivity measure. Below, we discuss the modeling approach for recognition memory measurement and why we chose to advocate for a scalar measure rather than a model-fitting method.

7 Notwithstanding measures that are linear transformations of each other, e.g., *P*_r_ and *P**ercent* *correct*.

8 For example, researchers that assume threshold models to mediate recognition performance may employ the formulas of *P*_r_ or *Percent* *correct* to compute sensitivity. Below we show that these measures assume a linear association between HRs and FARs. Likewise, researchers that assume signal detection models to mediate recognition performance typically employ formulas that are based on *z*-scores, such as the formula of *d*′. This measure implies a curvilinear association between HRs and FARs. For further explanation on this topic, see Pazzaglia et al., 2013; Rotello et al., 2015; Swets, 1986a, 1986b.

9 There are several techniques to get multiple points (e.g., asking participants to report their confidence levels; providing different payoffs for misses and for false alarms; see below for further explanation).

10 In an experiment that is low in power, it may be difficult to detect that two points obtained from iso-sensitive conditions do not fall on the same ROC curve, regardless of the measure of sensitivity that is used. However, with high-powered experiments, it will be easier to detect the discrepancy between the form of the ROC implied by the measure and the nature of the empirical ROC. For this reason, T1ERs increase as a function of an increase in sample size and in the number of trials (Rotello et al., 2008).

11 For the UVSD model and the double-high threshold models, sensitivity can be estimated with a known formula. This is not the case for the DPSD model, where modeling is required to obtain an estimate of sensitivity (no formula exists for DPSD to estimate sensitivity via a combination of HR and FAR or by using confidence ratings).

12 In our review of recognition memory articles, most authors that used mathematical modeling cite two R packages they used. An R package does not exist for the UVSD model (but there is a package in MATLAB). This might be the reason for the paucity of UVSD models in the modeling literature. Interested readers may consider using freely downloadable, general-purpose, signal detection software: https://hautus.org/sdt-assistant.php

13 It is possible to make parametric assumptions about the shape of the curve, specifically, to assume that the data are generated from Gaussian distributions. This measure is labeled *A*_z_, and its measurement model is completely equal to the measurement model of *d*_a_ (Hautus et al., 2022). In our review of the measures reported in the literature, not a single article included *A*_z_.

14 Note that for a Gaussian equal variance model, *S* = 1 and thus *d*_a_ reduces to *d*′.

15 This sampling procedure provides ideal conditions for demonstrating that a measure is bias-independent. Given identical observations, the same values of sensitivity ought to be obtained (within statistical error). Hence, if measures are found to *not* be valid under our data-sampling procedure, they would definitely not be valid when measuring sensitivity in real-world empirical data. The reverse is not true; measures that are found to be valid under those ideal conditions will still have to be tested in empirical settings to be endorsed as valid measures.

16 When running rectangular lure and target distributions, we wanted to cover the parameter space in a manner that is comparable to the Gaussian distributions (e.g., small and large distances between distributions have approximately the same meaning in regard to the lure SD). However, some distribution and criteria variables led to a very large exclusion of participants, such that in several simulations, we did not have enough participants to run a *t*-test. We therefore limited the experimental scenarios for rectangular distributions simulations. We still obtained results from 18 different simulations of rectangular distributions, each containing 10,000 iterations. All the results from those simulations appear in Appendix A.

17 Note that the degrees of freedom for the *t*-tests was not always equal to the number of simulated participants minus 1. In addition to the exclusion of participants explained in the method section based on the minimum three-points algorithm, some simulated participants yielded values of H=0% or FA=100%, reflecting erroneous responses for *all* target or lure trials, respectively. These participants too were excluded, so as to best mimic the analysis of empirical data. Indeed, in empirical setting, values of H=0% or FA=100% suggest that the participant did not understand task instructions or did not pay attention at either encoding or retrieval. Thus, such data would likely be excluded from analysis, a procedure we mimicked.

18 This was the case for all simulations where the size of shift in criteria between conditions was 0.5 or 0.7. However, for a very small shift in criteria (0.3), T1ERs sometimes reached values of “only” 98% or 99%. Because the small size of shift resulted in a small difference in bias, slightly fewer T1ERs occurred.

19 Because of the large number of simulations, idiosyncratic combinations of parameters sometimes yielded T1ERS higher than 5%. We did not document these cases because they did not seem to illuminate any pattern of conditions under which *d*_a_ could not be trusted to yield 5% error rates.

20 When exploring the parameter space in simulations, levels of different variables can create extreme experimental scenarios (see Discussion, Footnote 21, for an example). These scenarios are usually very unlikely in empirical settings. If a pattern of results is not observed across several levels of distributional and criteria variables, it should be taken with a grain of salt.

21 In the conservative placements, the middle criterion was set at 0.5. The mean of the lure and target distributions were set at equal distances from 0 (see Method). Therefore, in large-distance simulations (distance = 2 SDs relative to the lure distribution), described in Appendix F as the simulations that included the largest deviations of *S* from the SD ratio, the mean of the lure distributions was set at −1. If the mean of the lure distribution is −1, many signals sampled from this distribution are around −1. If the middle criterion (C3; see Method) was set at 0.5 for the liberal condition and shifted by 0.3–0.7 SDs (relative to the lure distribution; see Method) for the conservative condition, many signals will not exceed this criterion, resulting in many fewer “old” responses for lures. Ultimately, a large portion of participants will have a 0% FAR for at least one operating point. That is, they will have fewer operating points available for the computation of *S*. This was not the case for the liberal criteria placement where the middle criterion was placed at −0.5, but the mean of the lure and target distributions were like the conservative placement, therefore a larger portion of participants had all five operating points.
