# Supplementary material for: Reject common measures like d′ and Corrected Recognition, embrace da: Simulation explorations of single- and multi-point recognition measures of sensitivity
Source: Behav Res Methods. 2026 Sep 3;58(10):287. doi: 10.3758/s13428-026-03130-w (PMC13541828; doi:10.3758/s13428-026-03130-w)
Supplement: Supplementary file 1 [file 13428_2026_3130_MOESM1_ESM.docx]

**Reject common measures like *d*′ and *Corrected Recognition*, embrace *d*_a_: Simulation explorations of single- and multi-point recognition measures of sensitivity**

Adva Levi, Raz Danino, Caren M. Rotello and Yonatan Goshen-Gottstein

**Appendix C – Comparison of approaches for SD ratio estimation**

A goal of testing *d*_a_ in simulations was to obtain the best algorithm for estimating the SD ratio from the slope of the zROC curve (*S*), individually for each participant, per condition. The idea of obtaining a participant-level estimate rather than a group-level estimate (the mean estimate based on the entire sample, or based on the known SD ratio in the literature) was motivated by individual differences that are found within experiment in the estimate of the SD ratio, as we discuss in the introduction to the main text. The idea of requiring a minimum of three operating points was based on the notion that with only two operating points, the resultant *S* would be more influenced by sampling error than would be a slope derived from a minimum of three points. In this appendix we describe simulations designed to test the validity of this three-points algorithm as compared to four alternative algorithms for estimating the SD ratio.

Because the simulations described in the text of the article found the lowest T1ERs for *d*_a_ under the assumption of the UVSD model, we compared the four alternative algorithms under unequal-variance Gaussian data-generating models to determine which would yield approximately T1ERs of 5% consistently, across different experimental scenarios.

The four alternatives we tested fell into three categories. First, we tested an algorithm using a fixed value of the SD ratio. We used a lure-to-target SD ratio of 0.8, the mean estimate consistently reported in the literature (Mickes et al., 2007). Second, we tested two variations of algorithms of mean (sample-level) estimates of the SD ratio. In one variation, we computed one mean *S* across participants, based on the *z*ROC curves obtained across both simulated experimental conditions (liberal and conservative). Thus the same value of *S* was used for every participant in a given simulated experiment. In the other variation, we computed two mean *S* values across participants in a given experiment, one for the conservative condition and one for the liberal condition. We used the liberal mean *S* to estimate the SD ratio for each participant in the liberal condition, and the conservative mean *S* to estimate the SD ratio for each participant in the conservative condition. Third, we tested an alternative individual-level estimation of the SD ratio. Instead of the requirement of a minimum of three operating points on the zROC (per condition), we included participants with a minimum of two operating points in each condition.

All alternative algorithms shared an important characteristic that our main algorithm did not. For these algorithms, it was possible to obtain HR=100% or FAR=0%. Values of 0% and 100% cannot be converted to *z*-scores, and thus some form of correction method is required. In contrast, in our main algorithm, which required a minimum of three operating points per condition (i.e., our suggestion for computing *d*_a_), HRs and FARs are never equal to 100% and 0, so no correction was needed.

To clarify, under all alternative algorithms, some simulated participants’ responses resulted in 100% HR or 0% FAR for C3, and so corrections were required. For the set of simulations described in this appendix, we used the log-linear correction method. This choice of correction method was guided by previous simulations which found it to yield the lowest T1ERs, as compared to other correction methods (Rotello et al., 2008).

**Fixed parameter VS Individual three-points-minimum estimation**

Figure C1 depicts T1ERs of *d*_a_ using two different algorithms for estimating the SD ratio. The orange diamonds represent our proposed algorithm of individual-level estimation of the SD ratio based on zROC plots with minimum of three operating points per condition. The teal circles depict the results using a fixed value of *S* = 0.8 as the SD ratio for all participants in both conditions, with data simulated with three different data-generating models.

**Figure C1.** Type-I error rates of *d*_a_, using the three-points-minimum algorithm (orange diamonds), and the fixed value of 0.8 algorithm (teal circles), as a function of sample size. The figure includes simulations of equal-variance Gaussian lure and target distributions (left panel), unequal-variance Gaussian distributions with a population parameter of SD ratio = 0.8 (middle panel) and unequal-variance Gaussian distributions with a population parameter of SD ratio = 0.6 (right panel). The number of simulated trials was 128 trials per condition, the spread of criteria was neutral and wide, the distance between distributions was 1 SD (relative to the lure distribution), and the size of shift in criteria between conditions was 0.5 SD (relative to the lure distribution). The dashed line indicates α = .05.

**Min-3-points**

**Fixed value**

Our proposed algorithm of estimation at the participant level with a minimum of 3 operating points in both conditions resulted in approximately 5% T1ERs across all types of Gaussian simulations (Fig. C1, pink diamonds). However, the algorithm of choosing a fixed value yielded 5% T1ERs only when the data of each participant was simulated from distributions with a population SD ratio of exactly 0.8. When the distributions were equal variance T1ERs increase, as they also did when the population parameter of the SD ratio was 0.6, reaching 100% for larger samples (see Figure C1).

Importantly, in each of the three panels, the data of each and every participant was simulated from exactly the same distributions. This is not the case for empirical data, where each participant probably has memory strengths and a different SD ratio parameter, because the value of *S* varies across participants within the same experiment. Therefore, even given Gaussian distributions of unequal-variance, if a fixed value of SD ratio is used to compute *d*_a_, higher T1ERs will likely be found in empirical data for all participants with data-generating models that *deviate* from an SD ratio value of 0.8.

**Mean estimation VS Estimation at participant level**

Figure C2 depicts T1ERs of *d*_a_ using mean VS individual-level estimation of the SD ratio. The orange diamonds again represent our proposed algorithm, estimating the SD ratio based on zROC plots with minimum of three operating points per condition. The purple squares depict the T1ERs of *d*_a_ using the sample-level mean estimation of the SD ratio based on both conditions, and the grey triangles show T1ERs of da using the sample-level mean estimation of the SD ratio separately for each condition.

**Min-3-points**

**Mean-all**

**Mean-per-Condition**

64 trials

128 trials

256 trials

**Figure C2.** Type-I error rates of *d*_a_, using the three-points-minimum algorithm (orange diamonds), the sample-level mean-across-conditions algorithm (purple squares), and the sample-level mean-per-condition algorithm (grey triangles), as a function of sample size. The number of simulated trials was 64 (left panel), 128 (middle panel) or 256 (right panel). The data-generating model was unequal-variance Gaussian distributions (SD ratio = 0.8), the spread of criteria was neutral and wide, the distance between distributions was 1 SD (relative to the lure distribution), and the size of shift in criteria between conditions was 0.5 SD (relative to the lure distribution). The dashed line indicates α = .05.

Again, our proposed algorithm of individual estimations per condition yields in approximately 5% T1ERs across all sample sizes and number of trials. In contrast, when the mean S was used, either across conditions or separately for each condition, T1ERs were higher than 5% and increased as sample size increased. Interestingly, an increase in the number of trials resulted in lower T1ERs. Yet, even under the largest number of simulated trials (i.e., 256; right panel), T1ERs were higher than 5% under most experimental scenarios.

**Estimation with a minimum of two VS three operating points**

Our last comparison was the estimation of *S* at a participant level, using a minimum of two operating points as compared to a minimum of three points. Surprisingly, under most scenarios, the computation of *d*_a_ was identical under these two algorithms. In any given simulated experiment, if all participants had at least 3 operating points, then estimating *S* and computing *d*_a_ yielded identical results for the two algorithms. In the vast majority of our simulated experiments, that was the case.

We next considered experimental scenarios in which the median proportion of the sample size that was retained for analysis was at its lowest. (In these scenarios, criteria placement was conservative; see in the main text, Results, Figure 11). By definition, in these particular scenarios, the occurrence of participants with fewer than three operating points was relatively high. It was here that the difference between the two individual-estimation algorithms became apparent.

Figure C3 depicts T1ERs of *d*_a_ using the individual-level three-points-minimum S (orange diamonds), and *d*_a_ using the individual-level two-points-minimum *S* (teal circles), under conservative and wide criteria placements. The small simulated number of trials (i.e., 64 trials per condition) and the large shift in criteria (i.e., 0.7 SD relative to the lure) yielded a mean proportion of sample size retained for analysis (out of simulated sample size) of about 0.76 for all depicted sample sizes in Figure C3. Thus, on average, the median retained sample sizes were 19, 38 and 77 (for simulated sample sizes of 25, 50 and 100, respectively).

**Figure C3**. T1ERs of *d*_a_ using the individual-level three-points-minimum algorithm (orange diamonds), and the individual-level two-points-minimum algorithm (teal circles). The data was simulated from equal-variance Gaussian lure and target distributions (left panel) and unequal-variance Gaussian distributions with SD ratio of 0.8 (right panel). The number of simulated trials was 64 trials per condition, the spread of criteria was conservative and wide, the distance between distributions was 1 SD (relative to the lure distribution), and the size of shift in criteria between conditions was 0.7 SD (relative to the lure distribution). The dashed line indicates α = .05.

**Min-3-points**

**Min-2-points**

As we explained in the results (“*The effect of extent of participants' exclusion*”), under the scenarios depicted in the figure, T1ERs were higher than 5% even when using our three-points-minimum S for *d*_a_. However, using an algorithm based on a minimum of two points resulted in yet higher T1ERs. In this figure, for larger samples (N=100), the T1ERs using our three-points approach were approximately 15%, while T1ERs using minimum-two-points were higher than 30%.

**Summary.** From the five algorithms tested (our main algorithm and four alternatives), our strategy of estimating the SD ratio for each condition for simulated participants with a minimum of three operating points in *both* conditions, yielded the lowest T1ERs. Therefore, this is the algorithm we used throughout the simulations reported in the main text of the article. Note that this was also the only algorithm that avoided correction methods. Perhaps this contributed to the lower Type-I error rate that we observed.
