# Supplementary material for: Reject common measures like d′ and Corrected Recognition, embrace da: Simulation explorations of single- and multi-point recognition measures of sensitivity
Source: Behav Res Methods. 2026 Sep 3;58(10):287. doi: 10.3758/s13428-026-03130-w (PMC13541828; doi:10.3758/s13428-026-03130-w)
Supplement: Supplementary file 2 [file 13428_2026_3130_MOESM2_ESM.docx]

**Reject common measures like *d*′ and *Corrected Recognition*, embrace *d*_a_: Simulation explorations of single- and multi-point recognition measures of sensitivity**

Adva Levi, Raz Danino, Caren M. Rotello and Yonatan Goshen-Gottstein

**Appendix D – The Gaussian data-generating model**

As illustrated in the above figure, we simulated data from two distributions – Targets (i.e., memory strengths of studied, old, items) and Lures (i.e., memory strengths of unstudied, new, items). The number of signals sampled from each distribution (for each participant) was set to reflect the number of trials assumed in the simulation. We used the same signal values to calculate operating points in both the liberal and conservative bias conditions.

For example, if we simulated 128 trials per bias condition, then 64 were targets (random signals from the target distribution) and 64 were lures (random signals from the lure distribution), to be used for both conditions. To calculate five operating points in each bias condition, based on these signals, we compared the values of the signals to each criterion in the following way:

In each bias condition separately, we counted all the signals that exceeded C5 from the 64 targets and how many signals exceeded C5 from the 64 lures (note that each condition had different criteria). These signals reflect ‘old’ responses; the targets exceeding C5 reflecting ‘hits’ and the lures exceeding C5 reflecting ‘false alarms’. This step assumes that if participants were encouraged to make their most conservative decisions in the experiment, they would have labeled ‘old’ only the trials they responded to with a confidence rating of 6 (C5 is the most conservative criterion out of the five criteria).

Next, to simulate responses at a slightly lower level of confidence (i.e., a rating of 5 or 6), we counted as 'old' responses all the signals that exceeded C4, counting hits for signals from the target distribution that exceed C4, and false alarms for signals from the lure distribution that exceed C4. Here, we assumed that if participants made slightly less conservative decisions in the experiment, they would have labeled ‘old’ both the trials they responded to with a confidence rating of 6 and the trials they responded to with a confidence rating of 5.

The same logic can be applied to each criterion, therefore the same counting process was applied for all criteria in both conditions, resulting in five different counts of hits and false alarms for each condition, for each participant. (See Hautus et al., 2022, for additional details.)

Table D1 presents an example of a summary of this counting process for a situation in which there were 64 simulated target trials and 64 simulated lure trials in each bias condition:

**Table D1 – Example of trials count for 5 criteria**

|  | Liberal Condition | | Conservative Condition | |
| --- | --- | --- | --- | --- |
|  | Hits | False Alarms | Hits | False Alarms |
| C5 | 36 | 1 | 13 | 0 |
| C4 | 46 | 4 | 20 | 0 |
| C3 | 52 | 13 | 34 | 2 |
| C2 | 57 | 20 | 41 | 7 |
| C1 | 63 | 33 | 53 | 14 |

This example assumes signals were tallied against specific values of C1-C5 in each condition, and that C1-C5 were themselves defined relative to the values of the lure and target means. Concretely, if the mean of the lure distribution is 0, and we assume C1 in the liberal condition to be placed at 0, then C1 is placed at the mean of the lure distribution and thus, if signals are sampled randomly, approximately 50% of signals from this distribution will exceed C1. As you can see in this example (Table D1), 33 lure signals (out of 64) exceeded C1 in the liberal condition.

Based on the data in Table D1, operating points can be computed. The operating points require rates (as opposed to counts) of the hits and false alarms. To obtain rates, each count is divided by the total amount of targets or lures presented to the participant in each condition. In our simulation example, each condition included 64 targets and 64 lures, therefore each count of hits or false alarms had to be divided by 64. Table D2 includes the HRs and FARs, based on this example:

**Table D2 – Example of HR and FAR calculation, based on Table D1**

|  | Liberal Condition | | Conservative Condition | |
| --- | --- | --- | --- | --- |
|  | HR | FAR | HR | FAR |
| C5 | 0.56 | 0.02 | 0.20 | 0 |
| C4 | 0.72 | 0.06 | 0.31 | 0 |
| C3 | 0.81 | 0.20 | 0.53 | 0.03 |
| C2 | 0.89 | 0.31 | 0.64 | 0.11 |
| C1 | 0.98 | 0.52 | 0.83 | 0.22 |

In Table D2, each pair of HR and FAR constitute one operating point in that bias condition. In total, for this example, we have five operating points for the liberal condition and five operating points for the conservative condition. These operating points can now be transformed into z-scores, as long as the HR or FAR are not equal to 1 or 0. Table D3 includes the zHRs and zFARs, based on the presented example (note that for the conservative condition, where the FAR values were equal to 0, we present no z-scores for the entire operating point)-

**Table D3 – Example of zHR and zFAR calculation, based on Table D2**

|  | Liberal Condition | | Conservative Condition | |
| --- | --- | --- | --- | --- |
|  | zHR | Zfar | zHR | zFAR |
| C5 | 0.16 | -2.15 |  |  |
| C4 | 0.58 | -1.53 |  |  |
| C3 | 0.89 | -0.83 | 0.08 | -1.86 |
| C2 | 1.23 | -0.49 | 0.36 | -1.23 |
| C1 | 2.15 | 0.04 | 0.95 | -0.78 |

The values in Table D3 can be used as [*z*FAR, *z*HR] points to create a *z*ROC curve for each condition. In this example, we have five points for the liberal zROC and three points for the conservative *z*ROC. Using bivariate ordinary least-squares linear regression, we can compute a slope for each of the *z*ROC curves. In this example, the liberal slope is 0.84 and the conservative slope is 0.78. In our simulations, we used the individually-averaged slope for each simulated participant (i.e., for each participant, averaged across conditions) for the computation of *d*_a_. In this example, the average slope is *S* = 0.81.
