# Supplementary material for: Reject common measures like d′ and Corrected Recognition, embrace da: Simulation explorations of single- and multi-point recognition measures of sensitivity
Source: Behav Res Methods. 2026 Sep 3;58(10):287. doi: 10.3758/s13428-026-03130-w (PMC13541828; doi:10.3758/s13428-026-03130-w)
Supplement: Supplementary file 3 [file 13428_2026_3130_MOESM3_ESM.docx]

**Reject common measures like *d*′ and *Corrected Recognition*, embrace *d*_a_: Simulation explorations of single- and multi-point recognition measures of sensitivity**

Adva Levi, Raz Danino, Caren M. Rotello and Yonatan Goshen-Gottstein

**Appendix E - Sample size retained for analysis**

Correction methods were not required for our analyses because all cases wherein HR = 100% or FAR = 0% yielded fewer than three distinct operating points on the *z*ROC curve, resulting in the exclusion of the simulated participant. Therefore, in some iterations, when running 25, 50 or 100 participants per iteration, the sample size used to compute the *t*-test (i.e., the *sample size retained for analysis)* was smaller. The number of participants excluded from each simulation varied across types of simulations, as a function of number of trials and criteria placement. The table below presents a summary of the mean sample size retained for analysis, after the exclusions were carried out, for all Gaussian simulations.

| **Sample size retained for analysis** | 64 trials per condition | 128 & 256 trials per condition | All types of trials per conditions |
| --- | --- | --- | --- |
| Simulated N = 25 | 23.35 (0.54) | 24.83 (0.07) | 24.34 (0.23) |
| Simulated N = 50 | 46.80 (0.71) | 49.64 (0.11) | 48.69 (0.31) |
| Simulated N = 100 | 93.35 (0.98) | 99.25 (0.24) | 97.28 (0.48) |

We calculated the median and median absolute deviations (MAD) of the sample-size retained for analysis (post-exclusion) for each simulation. The mean of those medians (and MADs) for all simulations of 25, 50, and 100 participants, averaged across different number of trials, are presented in the table in Appendix E. In addition, the means of medians (and MADs) for simulations that used smaller number of trials (64 trials per condition) and for simulations that used larger number of trials (128 and 256 trials per condition), are also presented separately in this table.

Examination of the table reveals that for simulations with a small number of trials (i.e., 64 trials), a large number of participants were excluded relative to simulations with a higher number of trials (i.e., 128 and 256 trials). These results can be explained because in simulations with a small number of trials, fewer participants had a minimum of three operating points required for the estimation of *S*. Importantly, in such cases, fewer participants also had all five unique operating points. Over the course of the simulated experiments, having a high proportion of participants who were not excluded but still had fewer than the maximum of five operating points led to a less optimal estimation of the true SD ratio. Stated differently, in simulations with few trials, the estimate of *S* was based on lower-than-average number of operating points, thus yielding a less reliable estimate of the true population parameter. Accordingly, the Type 1 error rates for *d*_a_ increased. We return to this in the discussion.
