# Supplementary material for: Reject common measures like d′ and Corrected Recognition, embrace da: Simulation explorations of single- and multi-point recognition measures of sensitivity
Source: Behav Res Methods. 2026 Sep 3;58(10):287. doi: 10.3758/s13428-026-03130-w (PMC13541828; doi:10.3758/s13428-026-03130-w)
Supplement: Supplementary file 4 [file 13428_2026_3130_MOESM4_ESM.docx]

**Reject common measures like *d*′ and *Corrected Recognition*, embrace *d*_a_: Simulation explorations of single- and multi-point recognition measures of sensitivity**

Adva Levi, Raz Danino, Caren M. Rotello and Yonatan Goshen-Gottstein

**Appendix F - S values as estimations of the SD ratio**

An important aspect of *d*_a_ was the use of *S* as an estimate for the lure-to-target SD ratio (“SD ratio”). In our simulations, we had access to the true population SD ratios to which we could compare *S*. Table F1 presents the mean *S* values (and their standard errors, SE) across participants as a function of simulated SD ratio, criteria placements, number of trials, and distance between the lure and target distributions. To reiterate, in the Gaussian simulations, we ran lure-to-target SD ratios of 1, 0.8 and 0.6, with distances of 0.5, 1 and 1.5 SD relative to the lure distribution.

Examination of Table F1 reveals that overall, the mean values of *S* were approximately equal to the SD ratios assumed in our Gaussian data-generating models. Moreover, the variance of the estimates was lower as the number of trials increased (i.e., the SE of the mean slope was smaller for simulations with a higher number of trials). Note that the largest mean deviations from the true SD ratio were observed for conservative and wide placement of criteria.

A closer observation of the data revealed that for conservative and widely placed criteria, the largest deviations of *S* from the population SD ratio occurred in simulations comprising a small number of trials and large distances between the lures and target distributions. Table F1 shows that for Gaussian data-generating models with conservative and widely placed criteria, distance of 1.5 SD, and 64 trials, the mean *S* deviated from the simulated SD ratio by a magnitude of about 0.1-0.15. As we describe in Appendix E, when we simulated conservative criteria placements and a low number of trials (i.e., 64 trials), the retained sample size was the smallest, compared to the simulated sample size. We also describe that the smaller retained sample size is typically coupled with a zROC slope that is based on less operating points, thus producing a less accurate estimation of the SD ratio.

In summary, our investigation has uncovered experimental scenarios for which the value of *S* might be a less accurate estimate of the population SD ratio. First, a small number of trials might lead to a larger discrepancy between the measurement model and the data-generating model. In such cases, we expect *S* (as an estimate of the population SD ratio) to be less accurate. This pattern is indeed observed (see Table F1). Moreover, the criteria placement and distance between distributions might have an impact on the number of excluded participants, which can also reduce the accuracy of *S*.

**Table F1. Mean S values as a function of the data-generating model assumptions**

| **Criteria placement** | Distance between distributions (relative to the lure distribution) | Number of trials | EV Gaussian  (SD ratio=1) | UV Gaussian  (SD ratio=0.8) | UV Gaussian  (SD ratio=0.6) |
| --- | --- | --- | --- | --- | --- |
| Neutral & Wide | 0.5 SD | 64 | 1.018 (0.03) | 0.822 (0.03) | 0.616 (0.02) |
|  |  | 128 | 1.010 (0.02) | 0.808 (0.02) | 0.603 (0.02) |
|  |  | 256 | 1.003 (0.02) | 0.800 (0.01) | 0.599 (0.01) |
|  | 1 SD | 64 | 1.031 (0.04) | 0.765 (0.03) | 0.627 (0.02) |
|  |  | 128 | 1.017 (0.02) | 0.748 (0.02) | 0.610 (0.02) |
|  |  | 256 | 1.008 (0.02) | 0.736 (0.01) | 0.601 (0.01) |
|  | 1.5 SD | 64 | 1.045 (0.05) | 0.846 (0.04) | 0.641 (0.03) |
|  |  | 128 | 1.024 (0.03) | 0.828 (0.02) | 0.619 (0.02) |
|  |  | 256 | 1.014 (0.02) | 0.812 (0.02) | 0.607 (0.01) |
| Neutral & Narrow | 0.5 SD | 64 | 1.015 (0.05) | 0.810 (0.04) | 0.606 (0.03) |
|  |  | 128 | 1.003 (0.03) | 0.802 (0.03) | 0.601 (0.02) |
|  |  | 256 | 1.001 (0.02) | 0.800 (0.02) | 0.600 (0.02) |
|  | 1 SD | 64 | 1.023 (0.05) | 0.816 (0.04) | 0.610 (0.03) |
|  |  | 128 | 1.001 (0.03) | 0.799 (0.03) | 0.599 (0.02) |
|  |  | 256 | 0.998 (0.02) | 0.798 (0.02) | 0.598 (0.02) |
|  | 1.5 SD | 64 | 1.053 (0.06) | 0.841 (0.05) | 0.628 (0.04) |
|  |  | 128 | 1.007 (0.04) | 0.802 (0.03) | 0.600 (0.02) |
|  |  | 256 | 0.996 (0.03) | 0.796 (0.02) | 0.596 (0.02) |
| **Criteria placement** | Distance between distributions (relative to the lure distribution) | Number of trials | EV Gaussian  (SD ratio=1) | UV Gaussian  (SD ratio=0.8) | UV Gaussian  (SD ratio=0.6) |
| Liberal & Wide | 0.5 SD | 64 | 0.992 (0.03) | 0.805 (0.03) | 0.609 (0.02) |
|  |  | 128 | 0.992 (0.02) | 0.801 (0.02) | 0.599 (0.01) |
|  |  | 256 | 0.998 (0.02) | 0.800 (0.01) | 0.597 (0.01) |
|  | 1 SD | 64 | 0.988 (0.04) | 0.802 (0.03) | 0.611 (0.02) |
|  |  | 128 | 0.988 (0.02) | 0.801 (0.02) | 0.603 (0.02) |
|  |  | 256 | 0.933 (0.02) | 0.802 (0.01) | 0.599 (0.01) |
|  | 1.5 SD | 64 | 0.991 (0.05) | 0.802 (0.03) | 0.617 (0.02) |
|  |  | 128 | 0.986 (0.03) | 0.802 (0.02) | 0.609 (0.02) |
|  |  | 256 | 0.990 (0.02) | 0.804 (0.02) | 0.602 (0.01) |
| Liberal & Narrow | 0.5 SD | 64 | 1.023 (0.05) | 0.816 (0.04) | 0.609 (0.03) |
|  |  | 128 | 1.014 (0.03) | 0.808 (0.03) | 0.604 (0.02) |
|  |  | 256 | 1.006 (0.02) | 0.804 (0.02) | 0.602 (0.02) |
|  | 1 SD | 64 | 1.021 (0.05) | 0.821 (0.04) | 0.612 (0.03) |
|  |  | 128 | 1.020 (0.03) | 0.811 (0.03) | 0.605 (0.02) |
|  |  | 256 | 1.011 (0.02) | 0.805 (0.02) | 0.602 (0.02) |
|  | 1.5 SD | 64 | 1.002 (0.06) | 0.822 (0.04) | 0.616 (0.04) |
|  |  | 128 | 1.020 (0.04) | 0.814 (0.03) | 0.605 (0.02) |
|  |  | 256 | 1.017 (0.03) | 0.807 (0.03) | 0.602 (0.02) |
| **Criteria placement** | Distance between distributions (relative to the lure distribution) | Number of trials | EV Gaussian  (SD ratio=1) | UV Gaussian  (SD ratio=0.8) | UV Gaussian  (SD ratio=0.6) |
| Conservative & Wide | 0.5 SD | 64 | 1.048 (0.04) | 0.847 (0.03) | 0.636 (0.03) |
|  |  | 128 | 1.025 (0.03) | 0.824 (0.02) | 0.616 (0.02) |
|  |  | 256 | 1.012 (0.02) | 0.810 (0.02) | 0.606 (0.01) |
|  | 1 SD | 64 | 1.092 (0.04) | 0.877 (0.04) | 0.657 (0.03) |
|  |  | 128 | 1.044 (0.03) | 0.834 (0.02) | 0.624 (0.02) |
|  |  | 256 | 1.023 (0.02) | 0.817 (0.02) | 0.612 (0.01) |
|  | 1.5 SD | 64 | 1.154 (0.06) | 0.928 (0.05) | 0.695 (0.04) |
|  |  | 128 | 1.071 (0.03) | 0.855 (0.03) | 0.638 (0.02) |
|  |  | 256 | 1.035 (0.02) | 0.825 (0.02) | 0.617 (0.02) |
| Conservative & Narrow | 0.5 SD | 64 | 1.050 (0.06) | 0.839 (0.05) | 0.627 (0.04) |
|  |  | 128 | 1.006 (0.04) | 0.802 (0.03) | 0.600 (0.02) |
|  |  | 256 | 0.996 (0.03) | 0.795 (0.02) | 0.596 (0.02) |
|  | 1 SD | 64 | 1.102 (0.07) | 0.881 (0.06) | 0.660 (0.04) |
|  |  | 128 | 1.017 (0.04) | 0.812 (0.03) | 0.608 (0.03) |
|  |  | 256 | 0.993 (0.03) | 0.793 (0.02) | 0.595 (0.02) |
|  | 1.5 SD | 64 | 1.196 (0.08) | 0.956 (0.07) | 0.718 (0.06) |
|  |  | 128 | 1.064 (0.05) | 0.850 (0.04) | 0.637 (0.03) |
|  |  | 256 | 1.002 (0.03) | 0.801 (0.03) | 0.601 (0.02) |
